# Monitoring Changes to Alkenone Biosynthesis in Commercial *Tisochrysis lutea* Microalgae

**DOI:** 10.1021/acsomega.4c00087

**Published:** 2024-03-27

**Authors:** Gregory W. O’Neil, Allison Keller, Jazmine Balila, Sydney Golden, Nate Sipila, Britton Stone, Robert K. Nelson, Christopher M. Reddy

**Affiliations:** † Department of Chemistry, Western Washington University, Bellingham, Washington 98225 (United States); ‡ Department of Marine Chemistry and Geochemistry, Woods Hole Oceanographic Institution, Woods Hole, Massachusetts 02543, United States

## Abstract

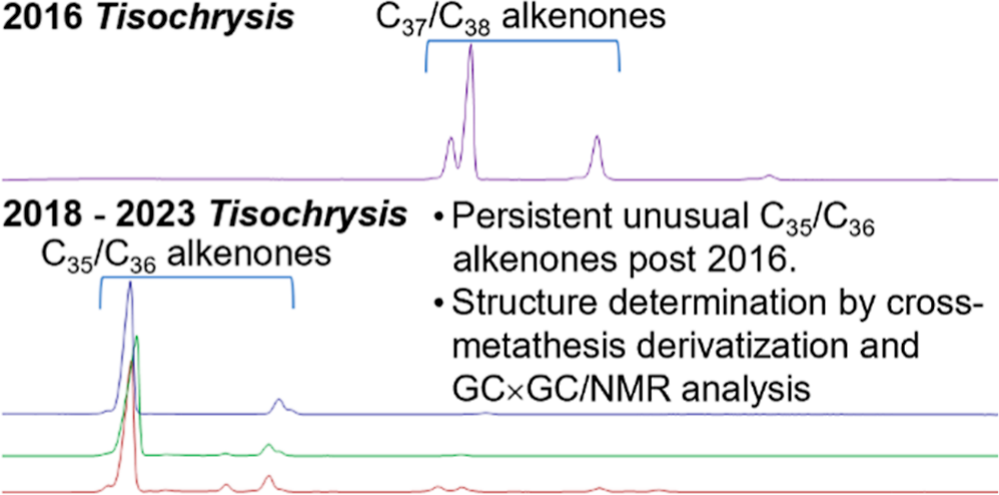

Alkenones are unique lipids produced by certain species of microalgae, well-known for use in paleoclimatology, and more recently pursued to advance sustainability across multiple industries. Beginning in 2018, the biosynthesis of alkenones by commercially grown *Tisochrysis lutea* (*T*-*Iso*) microalgae from one of the world’s most established producers, Necton S.A., changed dramatically from structures containing 37 and 38 carbons, to unusual shorter-chain C35 and C36 diunsaturated alkenones (C35:2 and C36:2 alkenones). While the exact reasons for this change remain unknown, analysis of alkenones isolated from *T-Iso* grown in 2021 and 2023 revealed that this change has persisted. The structure of these rare shorter-chain alkenones, including double bond position, produced by Necton *T-Iso* remained the same over the last five years, which was determined using a new and optimized cross-metathesis derivatization approach with analysis by comprehensive two-dimensional gas chromatography and NMR. However, noticeable differences in the alkenone profiles among the different batches were observed. Combined with fatty acid compositional analysis, the data suggest a connection between these lipid classes (*e.g.*, increased DHA corresponds to lower amounts of shorter-chain alkenones) and the ability to manipulate their biosynthesis in *T-Iso* with changes to cultivation conditions.

## Introduction

**Figure 1:**
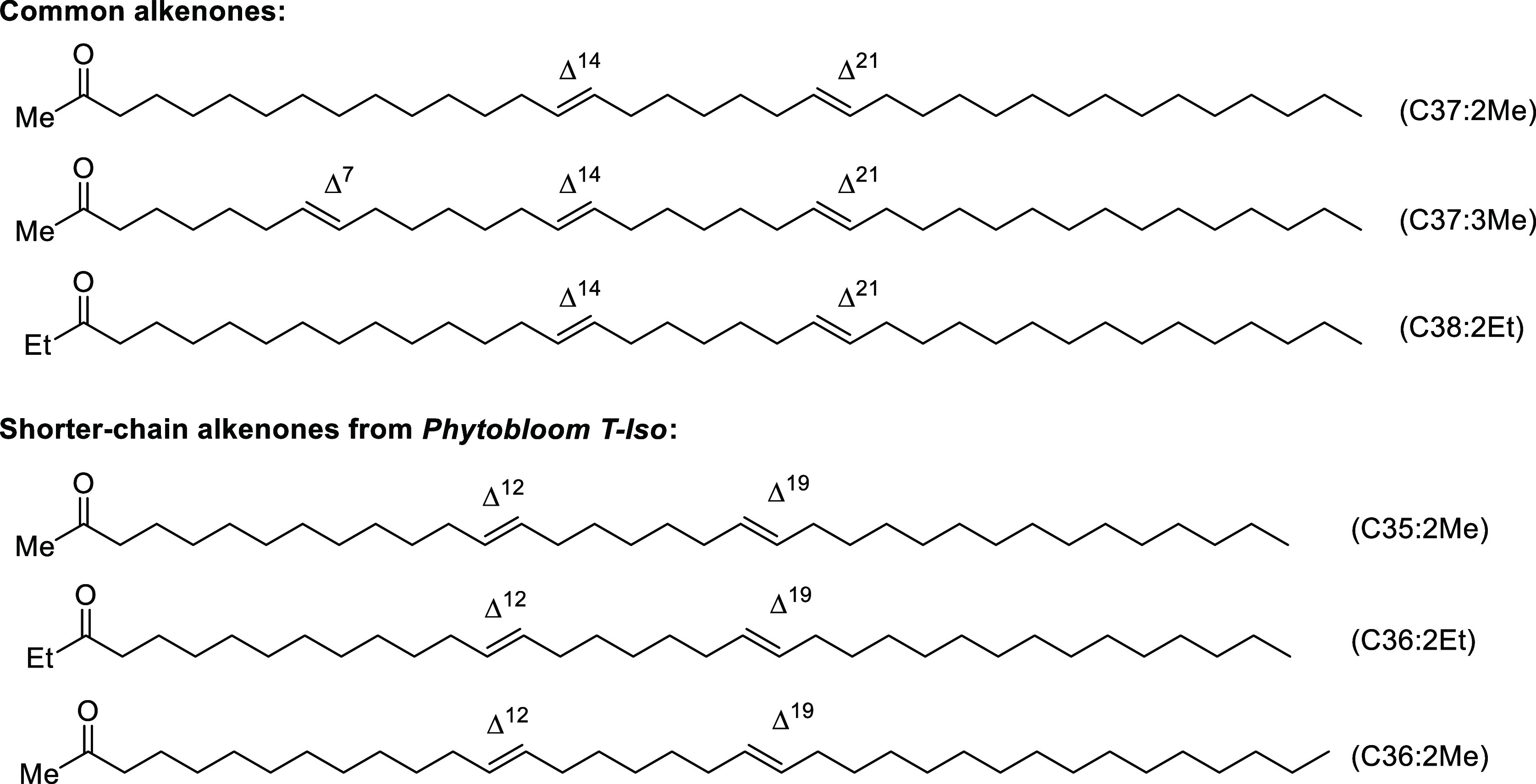
Structures of common alkenones (top) and rare shorter-chain alkenones isolated from commercial *Phytobloom T-Iso* produced since 2016.

*Tisochrysis lutea* (*T*-*Iso*)^1^ is a marine microalgae that has been cultivated for decades as a primary component of shellfish feed.^2^ More recently, new *T*-*iso* commercial applications have emerged, partly driven by global sustainability efforts.^3^ Examples include using *T-Iso* to produce high-value chemicals such as fucoxanthin and docosahexaenoic acid (DHA).^4−6^*T-Iso* is also one of a select few species of algae that biosynthesize a unique suite of neutral lipids known as polyunsaturated long-chain alkenones (alkenones).^7,8^ Alkenone structures are characterized by long hydrocarbon chains (typically C37–C40) containing 2–3 *trans*-double bonds and terminating in a methyl or ethyl ketone (Figure 1), making these compounds white waxes at room temperature (mp. ∼ 65 °C) and more stable than fatty acids (FAs) and other lipids with *cis* double bonds.^9^ The usage of alkenones found in marine sediments as paleoclimatological indicators is well established^10−14^ but yet to be fully explored as a sustainable material. Alkenones are now sold as a nonpetroleum-based waxy ingredient in personal care products^15^ and have promise in a variety of applications.^16−18^ One significant challenge for the successful commercialization of alkenones is consistent and predictable yields from *T-Iso* and other alkenone-producers.

Our group became interested in alkenones initially as part of investigations into the production of alternative fuels^19,20^ but have expanded to higher-value, lower-volume products such as phase change materials,^18^ cosmetics,^21^ and personal care products.^22^ We primarily purchased *T-Iso* from several suppliers in the US and Europe that consistently yielded alkenone mixtures containing common C37 and C38 alkenones and in some cases small amounts of C39 (Figure 1).^16−22^ Among our preferred *T-Iso* suppliers is Necton S.A. (Portugal), which has specialized in the commercialization of microalgae since 1997, making it Europe’s oldest company producing and selling microalgae.^23^ Necton’s *Isochrysis Phytobloom*(24) provided consistently good yields of alkenones and other lipids over nearly a decade of research. In 2018, however, unusual C35 and C36 alkenones were isolated as the major alkenones from a 4 kg batch of *Phytobloom* (Figure 1).^25^ After rigorous structure determination, we deduced that their structure matched those produced by the only other species of algae known to make primarily shorter-chain alkenones (*Emiliania huxleyi* CCMP2758). The observed shift in the composition and relative abundance of alkenones provide an opportunity to explore algae phylogeny and taxonomy,^26,27^ alkenone biosynthesis,^28^ and determine the impacts on paleothermometry and other alkenone-based technologies.

To determine if the C35 and C36 alkenones were an anomaly, we examined alkenones and FAs from *Phytobloom T-Iso* grown by Necton in 2021 and 2023. The results indicate that the switch to shorter-chain alkenones has remained, yet some noticeable changes to the alkenone profile have occurred. A new safer and further optimized cross-metathesis (CM)-based^29^ derivatization was used to determine alkenone double position, which proved identical for individual alkenones isolated from all of the samples (*e.g.*, separated by five methylenes). FA yields were comparable among the different *Phytobloom* products (7–12% w/DW algae), all mixtures characterized by high amounts of polyunsaturated fatty acids (PUFAs; ∼ 40–50% of total FAs). Yet DHA content was higher for the post-2016 shorter-chain alkenone producers (14–17.5 vs 11% from 2016 Phytobloom).

## Results and Discussion

### Alkenone and Fatty Acid Content in *Phytobloom T-Iso*

A comparison of different lots of *Phytobloom T-Iso* obtained (and/or grown) in 2016, 2018, 2021, and 2023 is presented in Table 1. Yields of oil extracted by Soxhlet with hexanes (Algal Oil) were similar for each (17–18% w/DW algae) and consistent with what we have obtained from other *T-Iso* sources.^16−20^ However, the more recent 2018, 2021, and 2023 *Phytobloom* algal oils contained significantly higher amounts of FAs (11–12 vs 7% for 2016) at the expense of alkenones (1.8–2.2 vs 3.4% for 2016). Overall, the FA profiles were similar, characterized by significant quantities of PUFAs, yet there are some notable differences (Table 2). For instance, the percentage of PUFAs was ∼10% less for the 2021 and 2023 batches compared to those of both the 2016 and 2018 products. Both the 2021 and 2023 batches still contained elevated levels of the valuable PUFA DHA (C22:6) compared to 2016 (1.9 and 1.8% *versus* 0.8%), but neither was quite as high as that measured for the 2018 sample (2.1%). Additionally, saturated FA (Sat. FA) content for the 2021 and 2023 algae were more similar to the 2016 *Phytobloom* than the 2018 material.

**Table 1:** Comparison of Product Yields from 2016, 2018, 2021, and 2023 Batches of *Phytobloom T-Iso* (Necton S.A.)^a^

product g (%w/DW)^A^	*2016 Phytobloom*	*2018 Phytobloom*	*2021 Phytobloom*	*2023 Phytobloom*
dry biomass	20	50	90	100
algal oil	3.6 (18%)	8.8 (18%)	15.0 (17%)	17.6 (18%)
fatty acids	1.4 (7%)	6.1 (12%)	9.9 (11%)	12.1 (12%)
DHA	0.15 (0.8%)	1.05 (2.1%)	1.71 (1.9%)	1.78 (1.8%)
neutral lipids	1.5 (7.5%)	1.9 (3.9%)	3.6 (4.0%)	3.9 (3.9%)
alkenones	0.7 (3.4%)	0.9 (1.8%)	2.0 (2.2%)	2.1 (2.1%)
C_35_ + C_36_		0.8 (1.6%)	1.9 (2.1%)	1.8 (1.8%)
C_37_ + C_38_	0.6 (3.3%)	0.03 (0.1%)	0.09 (0.1)	0.3 (0.3%)

a Notes for Table 1: ^A^Values listed are average amounts obtained in grams for two extraction/isolation events performed on the amount of dry biomass indicated. Numbers in parentheses are the percent yields of the different products relative to the starting dry biomass (% w/DW).

**Table 2:** Distribution of Fatty Acids Isolated from Commercial *Isochrysis Phytobloom* (*T. lutea*) Purchased in 2016, 2018, 2021, and 2023^a^

f**atty acid**	*2016 Phytobloom*	*2018 Phytobloom*	*2021 Phytobloom*	*2023 Phytobloom*
C14:0	19.4	13.4	19.5	19.4
C15:0	0.3	0.6	1.1	ND
C16:0	8.8	7.6	11.0	11.0
C16:1	5.5	4.2	5.2	2.0
C16:2	0.3	ND	ND	0.8
C16:3	0.5	ND	ND	0.5
C18:0	0.2	0.2	<0.1	0.9
C18:1^a^	14.3	7.8	8.1	14.3
C18:2	7.1	3.8	3.2	6.7
C18:3	13.5	8.2	6.3	7.7
C18:4	10.4	22.0	14.6	11.7
C18:5	3.0	ND	ND	ND
C20:5	ND	0.7	0.5	0.3
C22:5	2.0	0.1	<0.1	ND
C22:6	11.0	17.5	14.1	14.7
**PUFAs**^B^	**47.8**	**52.3**	**38.8**	**41.4**
**Sat. FAs**^C^	**28.7**	**21.8**	**31.6**	**31.3**

a Notes for Table 2. ^A^Values are relative percentages of each fatty acid (FA) listed among the total FAs within each sample. ^B^Combined Δ9 + Δ11 isomers. ^C^Defined as FAs with two or more double bonds. ^D^FAs containing no double bonds. ND = Not detected.

**Figure 2:**
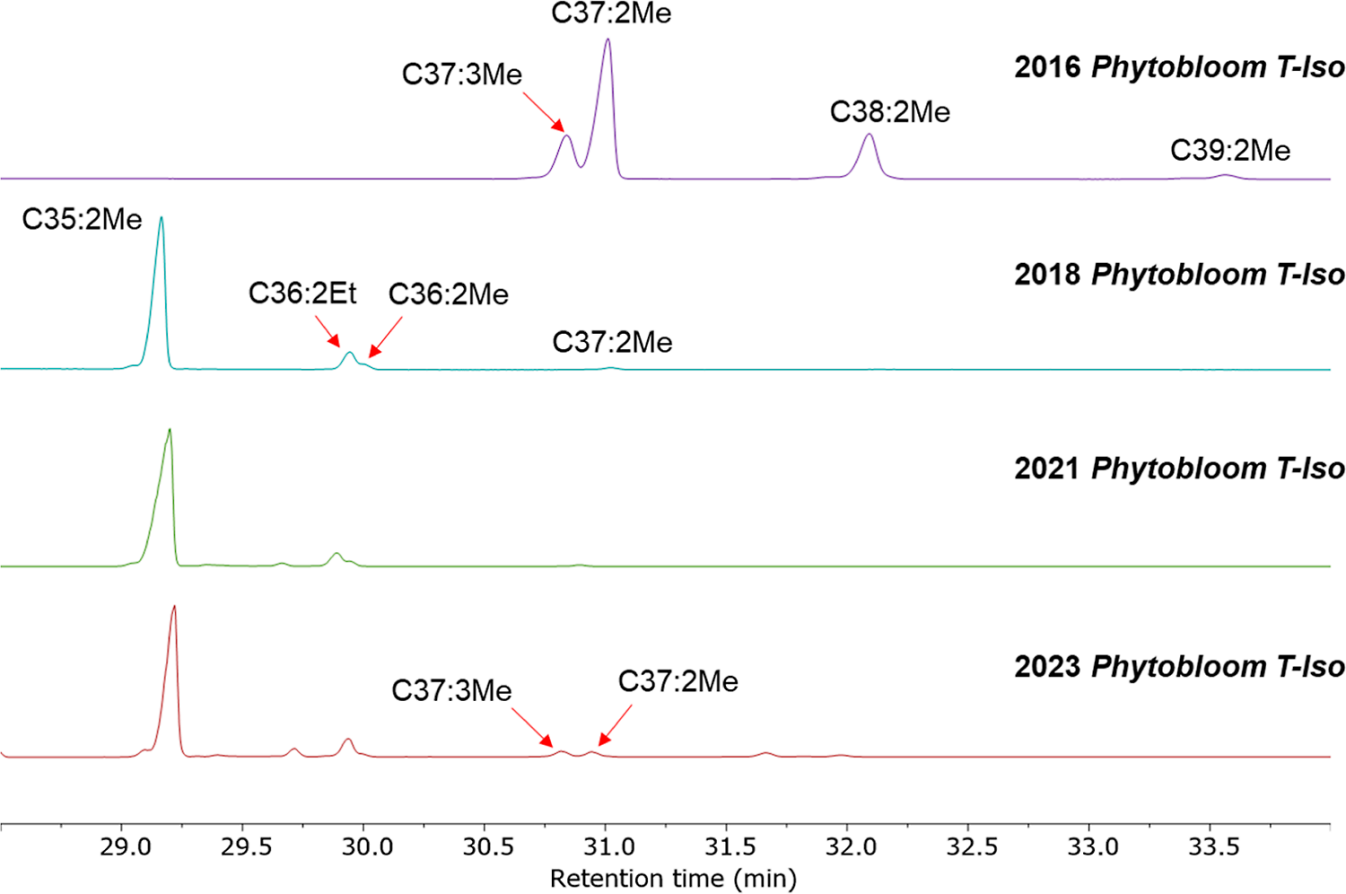
GC-FID chromatograms of *T-Iso* alkenones isolated from 2016 Necton *Phytobloom* (top), 2018 *Phytobloom* (middle), and 2021 *Phytobloom* (bottom), showing a dramatic shift toward shorter-chain C35/C36 alkenones between 2016 and 2018 that has persisted until 2023.

As shown by the GC-FID traces in Figure 2, alkenones isolated from the 2018, 2021, and 2023 *Phytobloom T-Iso* all consisted primarily of the same C35:2 and C36:2 alkenones [later confirmed by GC × GC-TOF HRMS and olefin metathesis derivatization (*vide infra*)], making them distinct from alkenone mixtures previously isolated by us^16−19^ and others.^30−32^ In fact, only one source of abundant C35/C36 alkenones is known,^33,34^ in this case a mutated strain of *E. huxleyi* (since deposited as a new strain CCMP2758 in the National Center for Marine Algae and Microbiota). Interestingly, alkenones from the 2023 *Phytobloom T-Iso* contained higher amounts of the more common C37 and C38 alkenones (7.4% total alkenones for 2023 *versus* 1.2 and 0.9% for 2018 and 2021, respectively) compared to the 2016 sample (Table 3). Like FAs,^35,36^ alkenone production by *Phytobloom T-Iso* (and likely other alkenone-producing microalgae) appears to be sensitive to changes that can occur during cultivation.^37^ The physiological role for alkenones is yet to be fully determined, yet they are likely used for energy storage.^38^ In this way, alkenones and FAs are related and therefore perhaps unsurprisingly their biosynthesis connected (*e.g.*, lower amounts of shorter-chain alkenones correlate with higher amounts of FAs including DHA).

**Table 3:** Comparison of Isolated Alkenones from Commercial *Isochrysis Phytobloom* (*T. lutea*) Purchased in 2016, 2018, 2021, and 2023^a^

alkenone^A^	*2016 Phytobloom*	*2018 Phytobloom*^B^	*2021 Phytobloom*^C^	*2023 Phytobloom*^C^
C35:2	ND	82.2	86.4	74.1
C36:2^A^	ND	12.9	8.4	9.7
C37:2Me	29	1.2	0.9	2.9
C37:3Me	46	ND	ND	3.5
C38:2Me	15	trace	trace	1.0
C38:2Et	5	ND	ND	ND
C39:2Me	3	ND	ND	ND
C39:3Me	2	ND	ND	ND

a Notes for Table 3: ^A^Percentages of alkenones listed according to GC-FID. Other trace components not listed are tentatively identified as alkenones or alkenoates. ^A^Combined Et and Me.

### Double-Bond Position of Alkenones

Aside from carbon chain length, tracking changes to alkenone production by microalgae also requires a determination of their double bond location. Resolving alkenone double bond positional isomers, however, is challenging and can require specialized instrumentation (*e.g.*, GC columns coated with optimized stationary phases^39,40^). Nonetheless, determining alkenone double bond position is a critical detail for not only understanding the role of external factors on alkenone biosynthesis but also their use in paleoclimatology and the development of new sustainable materials (*e.g.*, potential impact on physical properties or derivatives obtained by reactions at the double bonds).

**Scheme 1:**
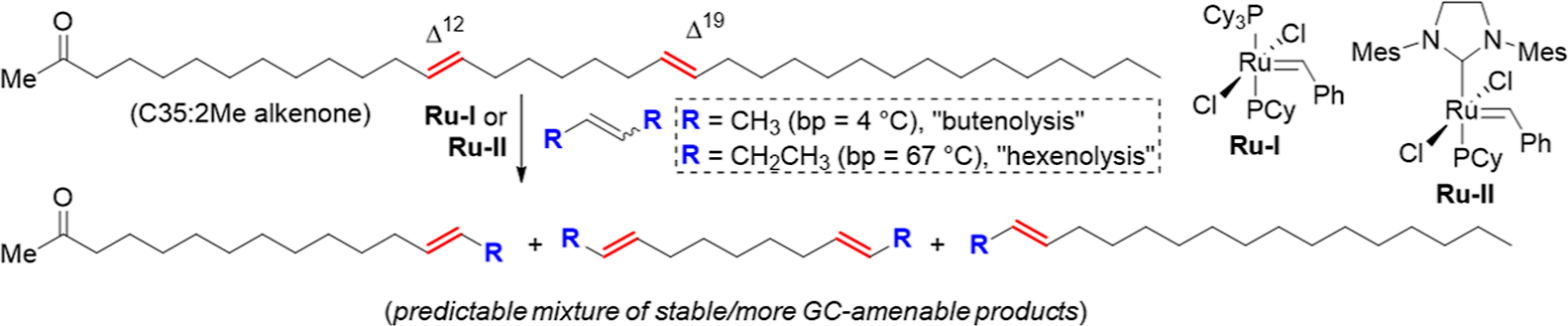
Comparison of Alkenone “Butenolysis” and “Hexenolysis” Both reactions produce a predictable mixture of products that can be used to identify double bond positions in the starting alkenone (*e.g.* C35:2 Me); however, hexenolysis avoids gaseous 2-butene making the reaction more approachable.

Previously, we used a CM reaction with 2-butene (“butenolysis”) to determine double bond positions when analyzing C35/C36 alkenones isolated from 2018 *Phytobloom T-Iso* (Scheme 1).^25^ This reaction effectively cleaves alkenones at their carbon–carbon double bonds, producing a predictable mixture of alkenone fragments from which the original alkenone structure can be deduced. Compared to other chemical methods for alkenone double bond position determination,^41−43^ butenolysis is attractive in giving high yields of more GC-amenable stable alkenone derivatives. However, the reaction requires 2-butene, a gas at room temperature (bp = 4 °C), raising possible safety concerns. Of course, one benefit is that any excess 2-butene evaporates readily, thereby not contaminating and complicating analysis of the butenolysis products.

Balancing the benefits and challenges of butenolysis, 3-hexene was identified as a potential alternative to 2-butene for CM reactions with alkenones (*i.e.*, “hexenolysis”) to determine their double bond positions. The boiling point of 3-hexene (67 °C) is low enough to be easily removed yet sufficiently high enough to be a liquid at room temperature. Moreover, like butenolysis, smaller/stable alkenone derivatives would be produced by hexenolysis, giving a mixture suitable for analysis by GC.

Hexenolysis was performed on alkenones isolated from 2021 *Phytobloom T-Iso* using Grubbs’ first- (**Ru–I**) and second-generation (**Ru–II**) catalysts^44^ and either *cis*- or *trans*-3-hexene in dichloromethane (DCM) at room temperature (Table 4). In general, the results reflect established metathesis reactivity trends.^29,45^ More specifically, the use of *cis*-3-hexene and **Ru–II** resulted in conversions comparatively higher than those of *trans*-3-hexene and **Ru–I**. For instance, using **Ru–II** and *cis*-3-hexene gave 81.7% conversion after 0.5 h, whereas this same reaction with **Ru–I** resulted in only 28.5% conversion of alkenones to metathesis products. Similarly, after 1 h, the combination of **Ru–II** and *cis*-3-hexene resulted in nearly complete conversion (95.7%), whereas only 50% conversion was obtained with *trans*-3-hexene under those same conditions.

**Table 4:** Results from Alkenone Hexenolysis Using Grubbs’ First- (**Ru–I**) and Second-Generation (**Ru–II**) Catalysts with Either *cis*- or *trans*-3-Hexene^a^

catalyst	3-hexene	time (h)	% conversion^A^	catalyst	3-hexene	time (h)	% conversion^A^
**Ru–I**	*cis*	0.5	28.5	**Ru–II**	*cis*	0.5	81.7
**Ru–I**	*cis*	1	35.3	**Ru–II**	*cis*	1	95.7
**Ru–I**	*cis*	3	64.2	**Ru–II**	*cis*	3	100
**Ru–I**	*cis*	6	76.3	**Ru–II**	*trans*	0.5	44.1
**Ru–I**	*cis*	15	90.4	**Ru–II**	*trans*	1	47.3
**Ru–I**	*trans*	0.5	25.6	**Ru–II**	*trans*	3	50.1
**Ru–I**	*trans*	1	28.1	**Ru–II**	*trans*	6	57.6
**Ru–I**	*trans*	3	31.3	**Ru–II**	*trans*	15	100
**Ru–I**	*trans*	6	38.1				

a Notes for table: all reactions were performed by dissolving alkenones (50 mg) in DCM (1 mL) and adding 3-hexene (0.2 mL) followed by catalyst (2 mg) and stirring for the time indicated. ^A^Percent conversions were determined by GC-FID by comparing the integration values for combined alkenones pre- and post hexenolysis. For those reactions reported as 100% conversion, no alkenone signal was detectable by GC-FID.

Since alkenone hexenolysis produced a complex mixture of products that was challenging to analyze by GC–FID/GC–MS, we opted to employ comprehensive two-dimensional gas chromatography (GC × GC). The increased resolution afforded by this technique has been successfully applied to the analysis of highly complex mixtures such as petroleum products,^46^ alkenone-containing extracts,^47^ and our previous alkenone butenolysis reactions.^25^ Coupled to a high-resolution time-of-flight mass spectrometer (TOF HRMS), the enhanced resolution and accurate mass data allow for a rigorous determination of alkenone structure.

**Figure 3:**
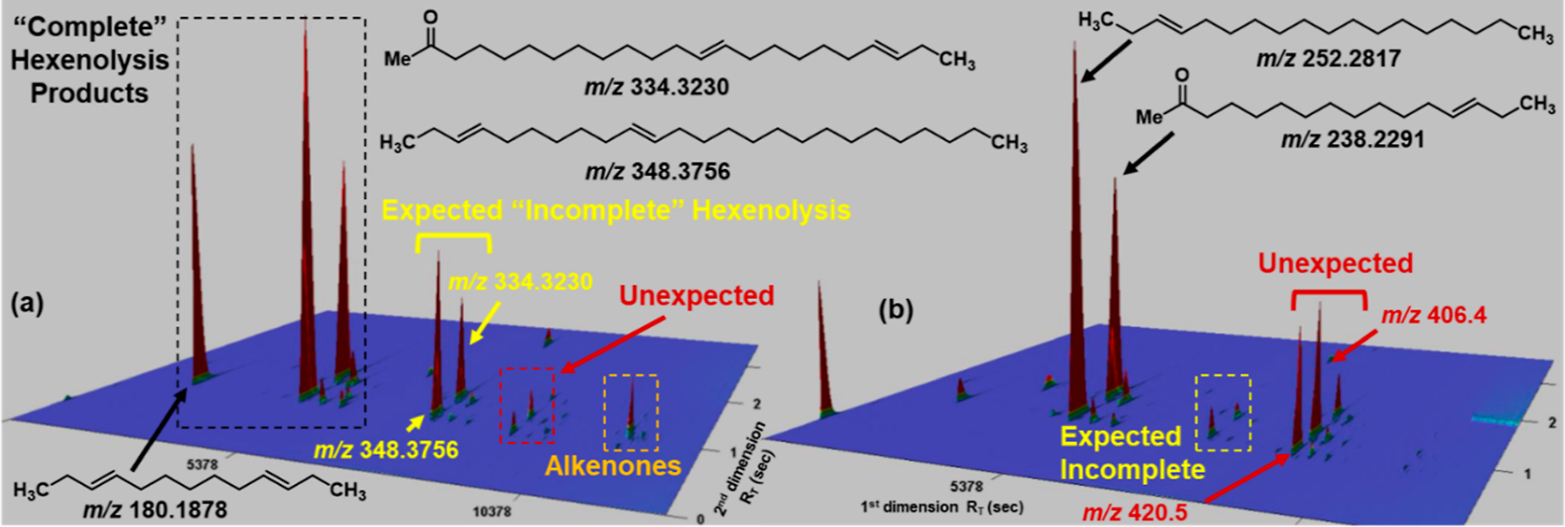
GC × GC-TOF mountain plot chromatograms of alkenone hexenolysis mixtures obtained using alkenones isolated from 2021 *Phytobloom T-Iso* performed with **Ru–I** [left, (a)] and **Ru–II** [right, (b)] under otherwise identical conditions (*trans*-3-hexene, rt., 15 h). “Incomplete” hexenolysis products [*e.g.*, (13*E*,20*E*)-tricosa-13,20-dien-2-one (*m*/*z* 334.3230) and (3*E*,10*E*)-pentacosa-3,10-diene (*m*/*z* 348.3756)] were more pronounced for the lower conversion reaction with **Ru–I** compared to “complete” hexenolysis products [*e.g.*, (3*E*,10*E*)-trideca-3,10-diene (*m*/*z* = 180.1878) and (*E*)-hexadec-13-en-2-one (*m*/*z* = 238.2291)]. A series of compounds with masses not readily assigned to expected hexenolysis products (*e.g.*, 406.4175 and 420.4695) were also detected that increased with increasing conversion (labeled “Unexpected”). Small peaks with similar second-dimension retention times and matching mass have been identified as *E*/*Z*-isomers (*e.g.*, *E*,*Z*- and *Z,Z*-isomers for dienes).^16^

As might be predicted, for those reactions with lower conversions, we observed greater amounts of “incomplete” hexenolysis products (*i.e.*, cleavage at only one of the double bonds for a di- or triunsaturated alkenone). For instance, from the reaction using *trans*-3-hexene and **Ru–I** after 15 h, (13*E*,20*E*)-tricosa-13,20-dien-2-one (*m*/*z* 334.3230) and (3*E*,10*E*)-pentacosa-3,10-diene (*m*/*z* 348.3756) were detected that were also produced in the same reaction with **Ru–II** (Figure 3). Still, these compounds could be considered “expected” products from incomplete hexenolysis and provide structural information about the starting alkenones. Interestingly, a few compounds with masses not readily assignable to hexenolysis products were found (*e.g.*, *m*/*z* 406.4169 and *m*/*z* 420.4695), and their contribution to the product mixture increased with increasing conversion.

**Scheme 2:**
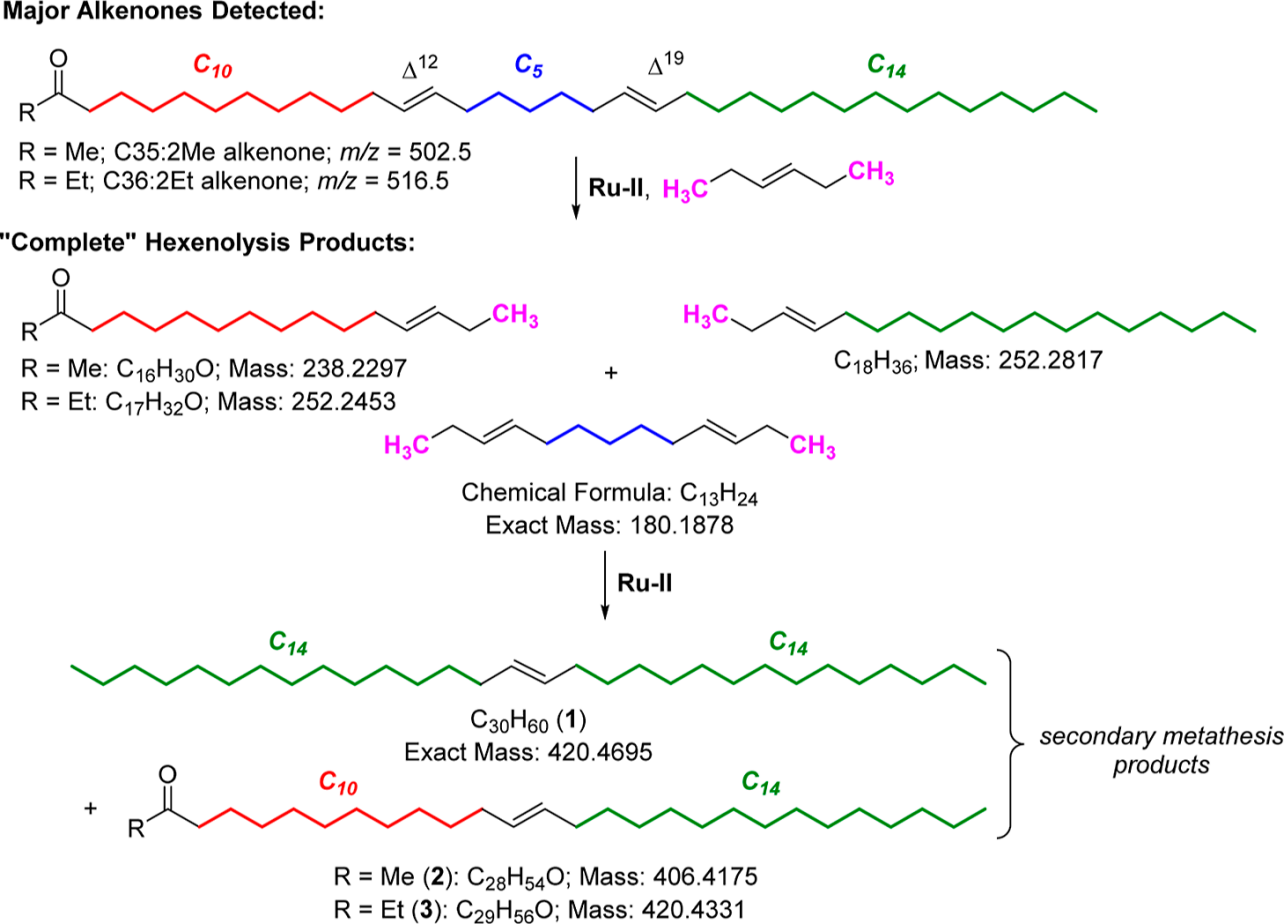
Further Reactions of “Complete” CM Products to Produce Unexpected Secondary Metathesis Products **1**, **2**, and **3** Detected in Alkenone Hexenolysis Product Mixtures

Ultimately these unexpected compounds were found to include (*E*)-triacont-15-ene (**1**), (*E*)-octacos-13-en-2-one (**2**), and (*E*)-nonacos-14-en-3-one (**3**), the result of initially formed hexenolysis products reacting further with themselves (Scheme 2).^48^ Despite using excess 3-hexene (>15 mol equiv), this secondary metathesis occurs to a significant extent, especially with the more reactive second-generation catalyst **Ru–II**.^49^ When analyzing alkenone butenolysis, products from secondary metathesis were not observed, presumably a reflection of the higher reactivity of 2-butene compared to 3-hexene in CM for steric reasons.^45^ While potentially interesting, for instance, as a pathway to generate new alkenone-based hydrocarbon mixtures of value for certain applications, secondary-metathesis complicates alkenone structure determination.

**Scheme 3:**
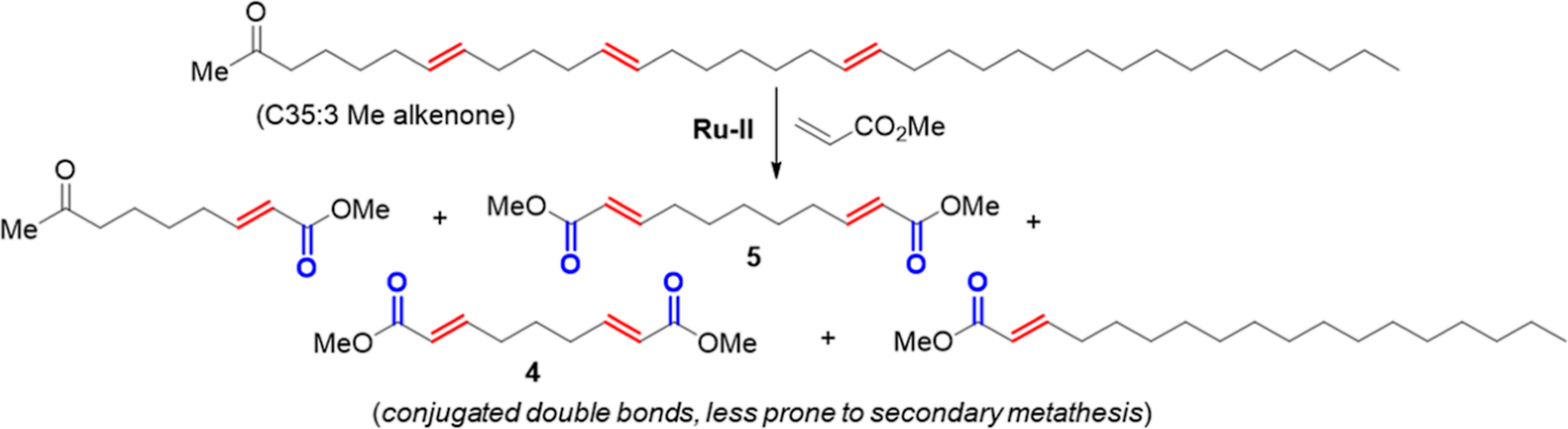
Representative Methyl Acrylate CM of a C35:3 Methyl Alkenone Containing Variable Carbon–Carbon Double Bond Spacing

Secondary metathesis was not observed during our previous studies on alkenone upgrading by CM with methyl acrylate (Scheme 3).^25^ In this case, the double bonds in the CM products are significantly different (*i.e.*, conjugated with C=O) than those in the starting alkenones and less prone to secondary metathesis,^47^ which is not the case for hexenolysis of CM products. As a result of these large structural differences between the starting alkenones and CM products, the progress of alkenone/methyl acrylate CM reactions can be tracked by ^1^H NMR.^18^ Additionally, acrylate CM reactions with lipids tend to be highly *trans*-selective,^50^ minimizing formation of geometric isomers and thereby simplifying analysis. We questioned if methyl acrylate CM might therefore represent an ideal method for interrogating alkenone double bond position, employing easy-to-handle reagents, avoiding secondary metathesis/geometric isomers, and allowing for analysis by routine NMR that does not require vaporization of high-molecular-weight alkenones and alkenone-derivatives.

**Figure 4:**
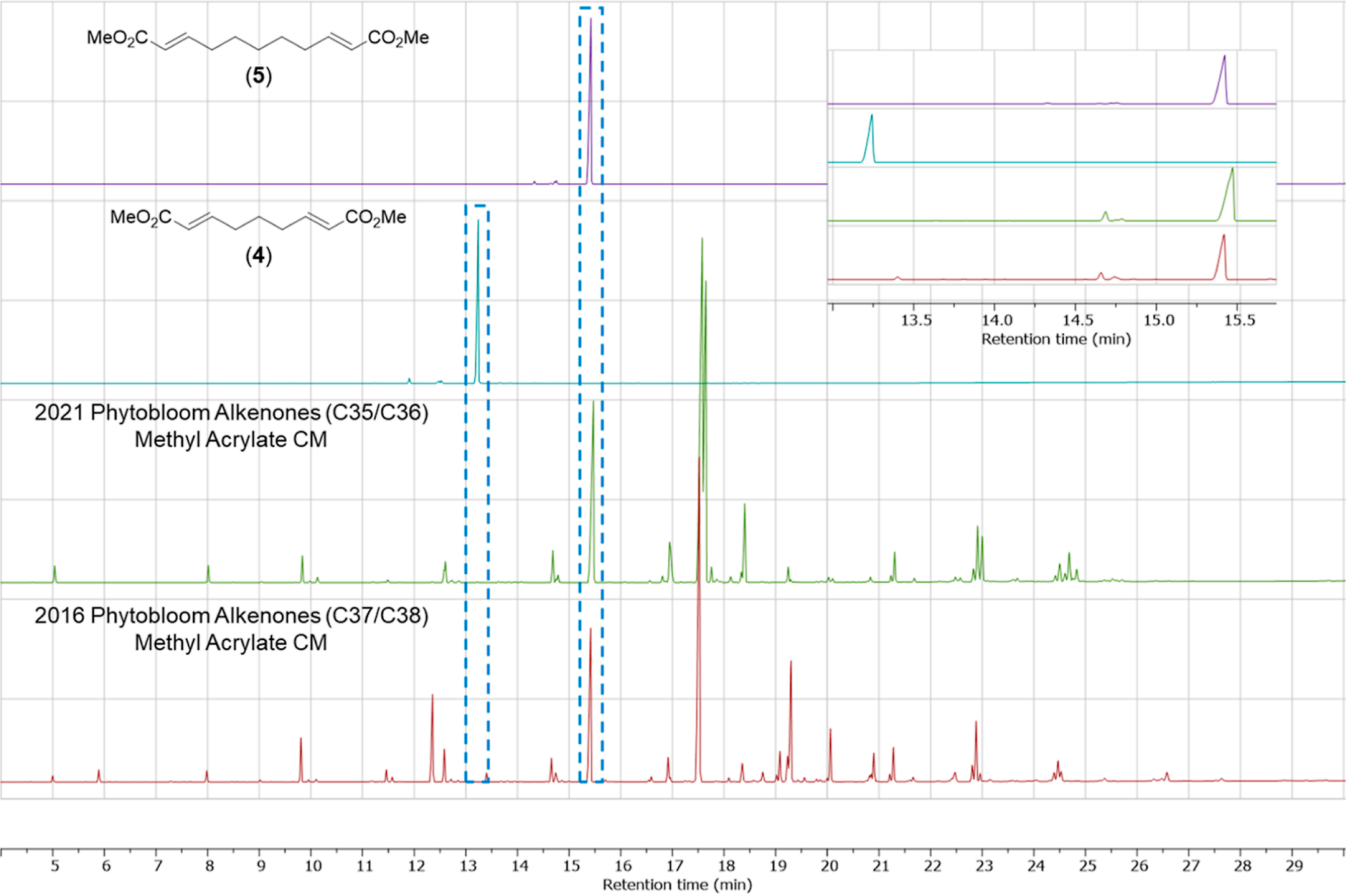
GC-FID chromatograms of methyl acrylate/alkenone CM product mixtures from 2021 and 2016 *Phytobloom Tisochrysis* (bottom) and standards **4** and **5** (top). Only compound **5** was detected from the CM reactions, indicating that alkenones contained only carbon–carbon double bonds separated by five methylenes.

**Figure 5:**
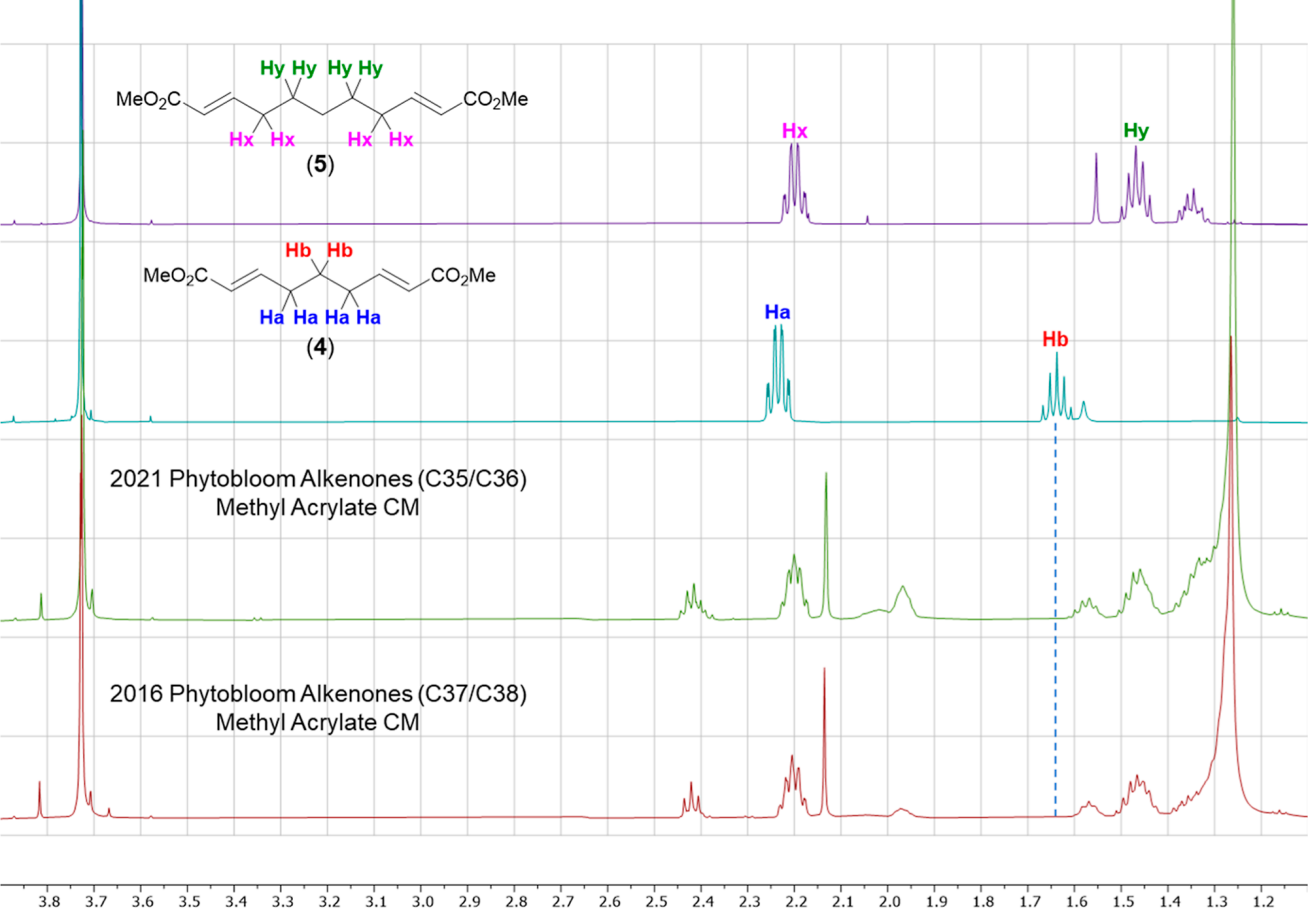
^1^H NMR spectra of methyl acrylate/alkenone CM product mixtures from 2021 and 2016 *Phytobloom Tisochrysis* (bottom) and standards **4** and **5** (top). Only compound **5** was detected (*e.g.*, see signal for Hb) from the CM reactions indicating alkenones only contained carbon–carbon double bonds separated by five methylenes.

To test this, samples of alkenones were treated with **Ru–II** in the presence of methyl acrylate. Additionally, dimethyl (2*E*,7*E*)-nona-2,7-dienedioate (**4**) and dimethyl (2*E*,9*E*)-undeca-2,9-dienedioate (**5**) were synthesized separately^51^ to be used as standard references representing the expected CM products from alkenones containing double bonds separated by three- or five-methylenes, respectively (*ref*. Scheme 3). As can be seen, using both GC-FID (Figure 4) and ^1^H NMR (Figure 5), it is clear from this method that *Phytobloom T-Iso* makes exclusively alkenones containing only double bonds separated by five methylenes.

## Conclusions

Altogether, the major alkenones produced by *Phytobloom T-Iso* continues to be (15*E*,22*E*)-heptatriaconta-15,22-dien-2-one and (16*E*,23*E*)-octatriaconta-16,23-dien-3-one since the switch between 2016 and 2018. It is noted that the EU’s “Microalgae As a Green source for Nutritional Ingredients for Food/Feed and Ingredients for Cosmetics by cost-Effective New Technologies” (MAGNIFICENT) project was launched during this window,^52^ which included strain development of *T-Iso* by Necton and its partners, for instance toward higher DHA production. In addition to increasing DHA, our results indicate that the changes implemented greatly affected alkenone biosynthesis, causing lower quantities of unusual C35/C36 to be produced that have persisted for the last five years. Minor differences in the alkenone composition from the most recently grown algae, in particular the re-emergence of more common C37 alkenones, could signify new modifications at the cultivation stage. Efforts toward identifying those parameters combined with genetic level analysis of the different crops are currently underway and will be reported in due course. The results could have important implications for alkenone commercialization efforts (*e.g.*, optimizing alkenone production) as well as a fundamental understanding of their physiological role and factors affecting alkenone biosynthesis.

## Methods

### General

NMR spectra were recorded on a Bruker 500 MHz spectrometer in CDCl_3_ as a solvent. GC-FID: Agilent 7890. GC × GC-TOF: Leco Pegasus 4D equipped with a Hewlett-Packard 6890 GC (TOFMS) and 7890 GC (FID system). DCM for CM reactions was dried by passing the solvent through a column of activated alumina under nitrogen. Methyl acrylate was distilled prior to use. Other reagents were purchased and used as received unless otherwise mentioned: solvents (Fisher Scientific, ACS Certified), 3-hexene (TCI Chemicals, >95% purity), Grubbs catalysts (Sigma-Aldrich). TLC analysis used 0.25 mm silica layer fluorescence UV_254_ plates. Column chromatography: silica gel (230–400 mesh).

### Microalgae

2 kg each of *T-Iso* (sold as *Isochrysis Phytobloom*) was received from Necton S.A. (Olhão, Portugal) that had been grown in 2021 (batch no. L3210418) and 2023 (batch no. L3210422). The algae were received as a dry milled powder that was light brown in color.

### Extraction and Isolation of Alkenones and Fatty Acids

Alkenones and FAs were isolated and purified from the 2021 and 2023 *Phytobloom T-Iso*, as previously described.^25,53^ Briefly, extraction with hexanes by Soxhlet produced a dark green near-black oily solid called algal oil. The latter was then redissolved in methanol/DCM (2:1, 10 × volume of algal oil) and treated with KOH (50% w/w) at 60 °C for 3 h. The resulting saponified acylglycerols were selectively partitioned into water, and the alkenone-containing neutral lipids were extracted into hexanes. Removal of the hexanes provided the neutral lipids as a red-brown solid, from which alkenones could be isolated by recrystallization. The aqueous solution containing the saponified acylglycerols was re-acidified with HCl (6 M), and the resulting FAs were then extracted into hexanes. Removal of the hexanes produced FAs as a dark green/brown liquid. Samples were stored frozen at −20 °C prior to analysis to prevent PUFA degradation.^54^

### Determination of Fatty Acids

The FA profile of isolated FAs was determined by Exact Scientific Services (Ferndale, WA) according to AOCS Methods Ce 2–66 (Preparation of Methyl Esters of Fatty Acids), Ce 1 × 10^–91^ (Determination of Fatty Acids in Edible Oils and Fats by Capillary GLC), and Ce 1b-89 Fatty Acid Composition by GLC). Briefly, FAs were converted to FA methyl esters (FAMEs) with a solution of BF_3_ in methanol. FAMEs were then extracted with heptane and analyzed *via* GC-FID [column: Restek (State College, PA) RT-2560 100 m; constant flow: 1 mL/min split: 10:1; oven: initial temp 100 C (hold 4 min), ramp rate 3 C/min to final temp 240 C (hold 15 min)]. Percent composition was determined by area % of each FA in relation to the total area % of sum of all FAs.

### Analysis by ^1^H NMR Spectroscopy

Nuclear magnetic resonance (^1^H NMR) spectra were obtained on a Bruker 500 MHz instrument under ambient conditions using CDCl_3_ as the solvent, which also served as an internal reference (a shift value of residual proton at 7.26 ppm).

### Analysis by One-Dimensional Gas Chromatography with Flame Ionization Detection

Purified alkenones were analyzed on a gas chromatograph with flame ionization detection (GC-FID). Samples (1 μL) were injected cool-on-column and separated on a 100% dimethyl polysiloxane capillary column [Agilent (Wilmington, DE) HP-5, 30 m length, 0.32 mm I.D., 0.25 μm film thickness] with He as the carrier gas at a constant flow of 6.5 mL min^–1^. The GC oven was programmed from 75 °C (0.5 min hold) and ramped at 2 °C min^–1^ to 320 °C (5 min hold).

### Alkenone CM

Alkenone CM reactions were performed by dissolving alkenones (50 mg) in dry and degassed DCM (5 mL) at room temperature under a N_2_ atmosphere, followed by the addition of 3-hexene (0.2 mL) or methyl acrylate (0.1 mL) and the Grubbs’ catalyst (2 mg). The mixture was stirred at room temperature for 0.5–15 h before concentrating on a rotary evaporator and analyzing by GC-FID, NMR, and GC × GC.

### Analysis by Comprehensive Two-Dimensional Gas Chromatography and High-Resolution Time-Of-Flight Mass Spectrometer

Purified alkenones and CM reaction mixtures were analyzed by two-dimensional gas chromatography and high-resolution time-of-flight mass spectrometer (GC × GC-TOF HRMS) according to previously described methodologies (Supporting Information).

### Synthesis of Compounds **4** and **5**

These are known compounds and were synthesized according to the method of Randl:^51^ To a solution of cyclopentene (0.36 mL, 3.95 mmol) or cycloheptene (0.46 mL, 3.95 mmol) in DCM (13 mL) was added methyl acrylate (0.24 mL, 2.62 mmol) followed by **Ru–II** (0.17 g, 0.2 mmol), and the mixture was stirred at 45 °C for 6 h under a nitrogen atmosphere. After cooling to room temperature and concentrating on a rotary evaporator, the crude product was purified by chromatography on silica (4:1 hexanes ethyl acetate) to yield **4** (0.42 g, 75%) or **5** (0.50 g, 79%) as colorless oils. Spectral data matched that previously reported for dimethyl (2*E*,7*E*)-nona-2,7-dienedioate (**4**)^55^ and dimethyl (2*E*,9*E*)-undeca-2,9-dienedioate (**5**).^56^

## Supporting Information Available

Description of GC × GC-TOF HRMS method, GC × GC-TOF chromatograms, and mass spectra for alkenone hexenolysis products, and NMR spectra of compounds **4**, **5**, and alkenone acrylate CM product mixtures (PDF)
