# Supplementary material for: Monitoring Changes to Alkenone Biosynthesis in Commercial Tisochrysis lutea Microalgae
Source: ACS Omega. 2024 Mar 27;9(14):16374–83. doi: 10.1021/acsomega.4c00087 (PMC11007839; doi:10.1021/acsomega.4c00087)

Supplementary Material for

Monitoring Changes to Alkenone Biosynthesis in Commercial Tisochrysis Lutea Microalgae

Gregory W. O'Neil,*[,a] Allison Keller,[a] Jazmine Balila,[a] Sydney Golden,[a] Nate Sipila,[a] Britton Stone,[a] Robert K. Nelson,[b] and Christopher M. Reddy[b]

[a] *Department of Chemistry, Western Washington University, Bellingham, WA, 98225 (USA)*

[b] *Department of Marine Chemistry and Geochemistry, Woods Hole Oceanographic Institution, Woods Hole, MA, 02543 (USA)*

Contents

GC×GC methods description	S1
**Figure S1.** GC×GC chromatograms of alkenone hexenolysis product mixtures.	S2-S5
**Figure S2.** GC×GC-TOF High Resolution Mass Spectra of intact alkenones and hexenolysis products.	S6-S10
**Figure S3.** ^{1}H NMR spectra of compounds **4**, **5**, and alkenone acrylate CM product mixtures.	S11-S12

Analysis by comprehensive two-dimensional gas chromatography and time of flight mass spectrometer (GCxGC-TOF). Two Leco Pegasus 4D GC×GC systems were used in this study coupled with a TOFMS and a FID, respectively. They were equipped with a Hewlett-Packard 6890 GC (TOFMS) and a 7890 GC (FID system) and configured with split/splitless auto-injectors (7683B series) and a dual stage cryogenic modulator (Leco, Saint Joseph, Michigan). Samples were injected in splitless mode. The modulator operates with a cold and hot jet. The cold jet gas was dry N_2, chilled with liquid N_2. The hot jet was operated with air that was heated at 5 °C above the temperature of the main GC oven. Two capillary GC columns were fitted in each GC×GC instrument. The first-dimension column was a non-polar Restek Rxi-1ms, (60 m length, 0.25 mm I.D., 0.25 µm film thickness) and the second-dimension separations were performed on a 50% phenyl polysilphenylene-siloxane column (SGE BPX50, 1.0 m length, 0.10 mm I.D., 0.1 µm film thickness).

For GC×GC-TOF analysis, the temperature program of the main oven started isothermal at 45 °C (10 min) and was then ramped from 100 to 340 °C at 1.50 °C min^{-1}. The hot jet was pulsed width was 1.0 and the modulation period was 6.0 seconds with a 2.00 second cooling period between stages. The second-dimension oven was programmed from 105 °C (10 min) to 345 °C at 1.50 °C min^{-1}. The TOFMS data were sampled at an acquisition rate of 100 spectra per second. The transfer line from the second oven to the TOFMS was deactivated fused silica (0.5 m length, 0.18 mm I.D.), constantly held at 315 °C. The TOF detector voltage was 1335 Volts and the source temperature 220 °C. The mass spectrometer employs 70 eV electron ionization and operates at a push pulse rate of 5 kHz allowing sufficient signal averaging time to ensure good signal-to-noise ratios while still operating at a high enough data acquisition rate to accurately process (signal average) spectra from the peaks eluting from the second-dimension column in this high resolution separation technique with second dimension peak widths on the order of 50 to 200 milliseconds.

Figure S1. Total ion chromatograms (plan view (top) and mountain plot (bottom)) from GC×GC analysis of alkenone hexenolysis product mixtures.

- Ru-I, *trans*-3-hexene, DCM, rt, 15 h.

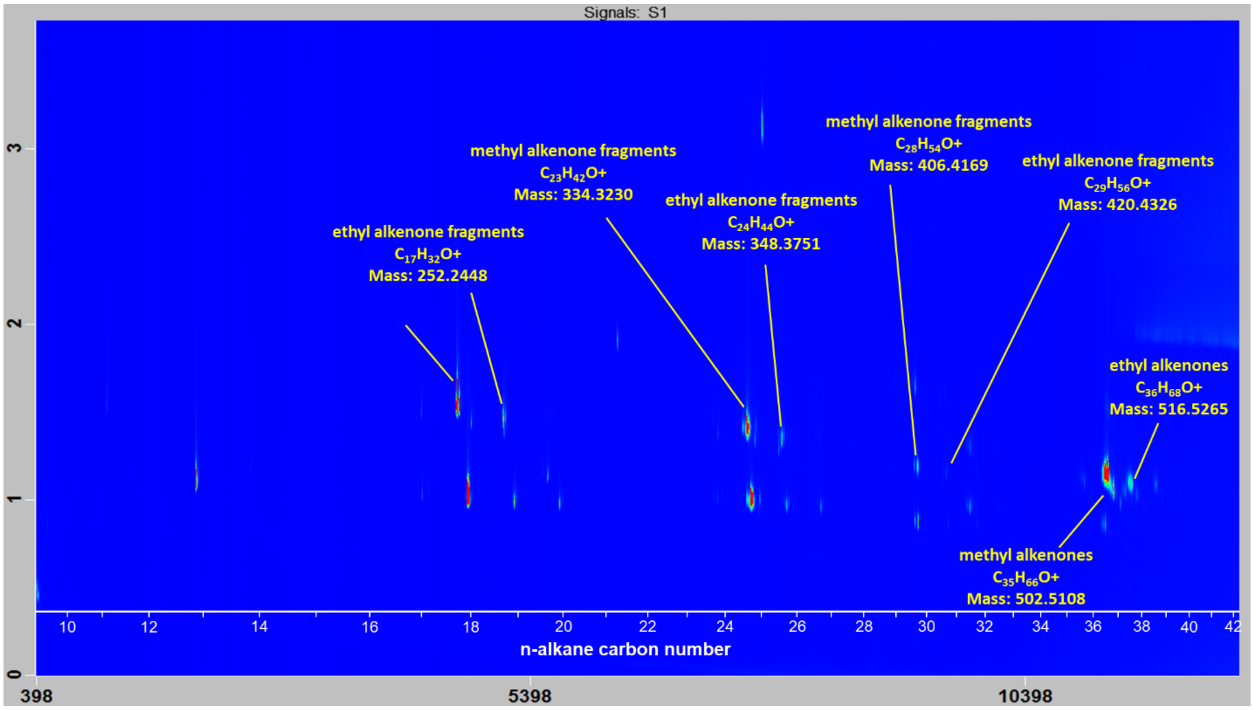

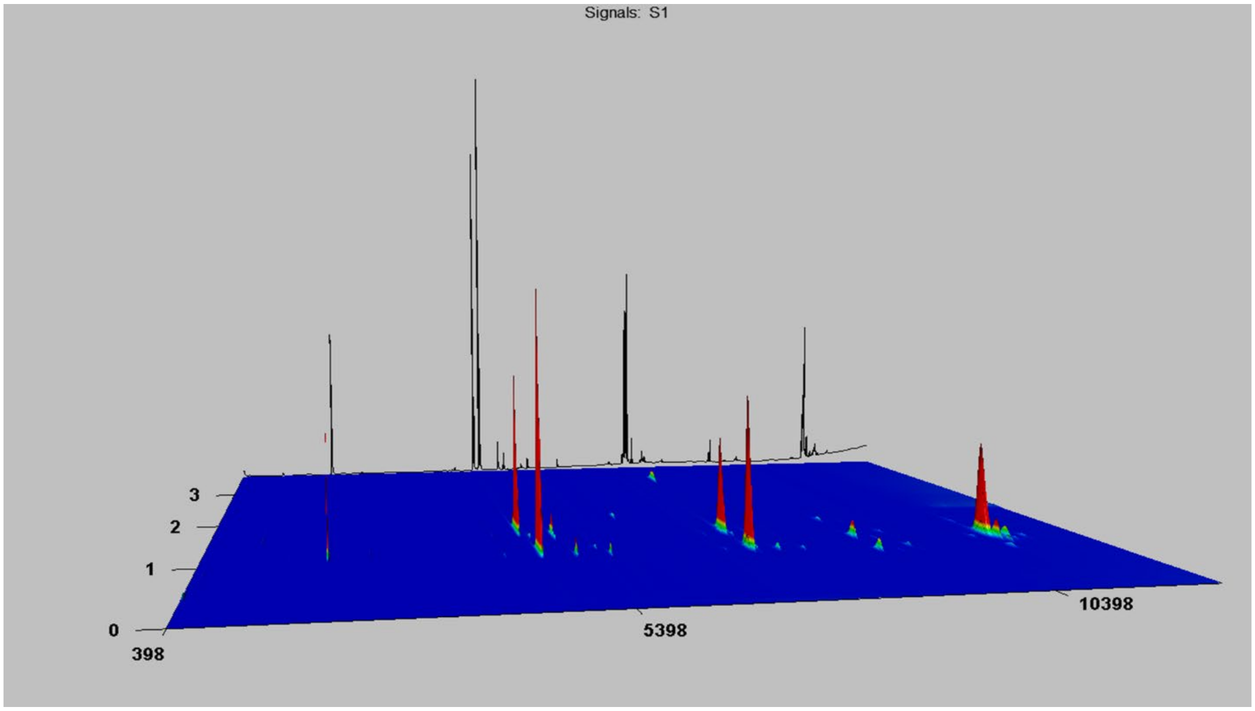

- Ru-I, *cis*-3-hexene, DCM, rt, 15 h.

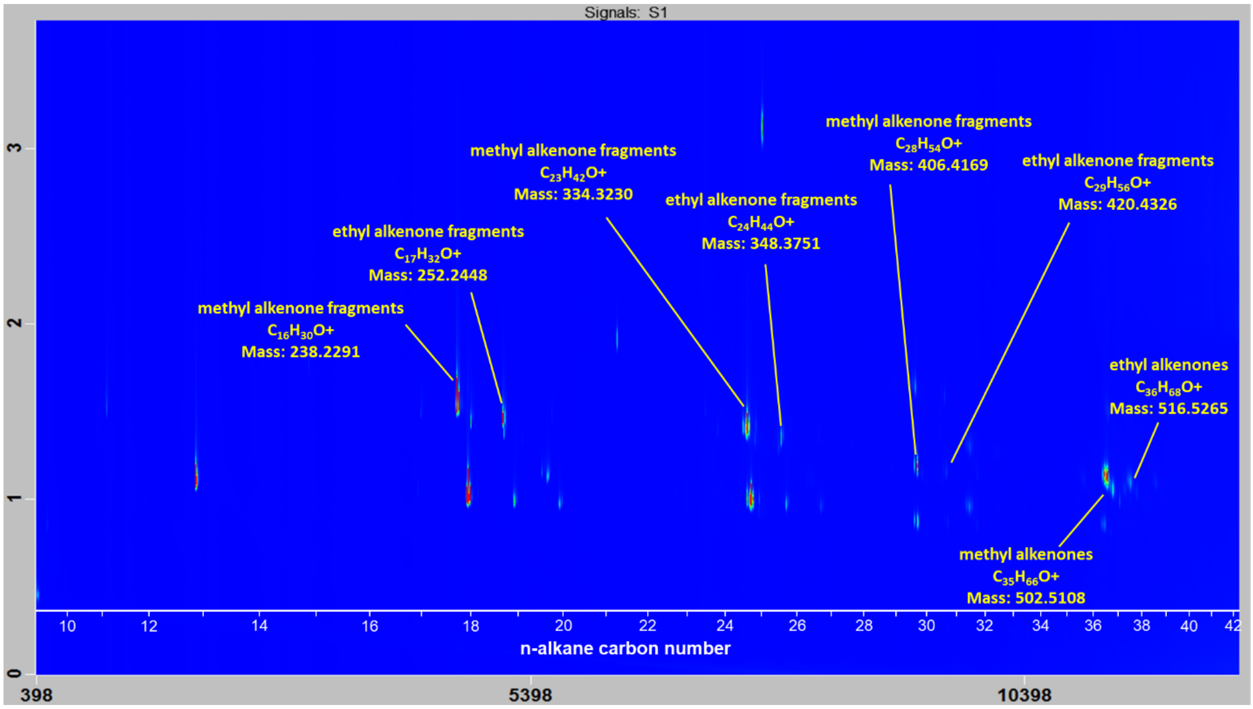

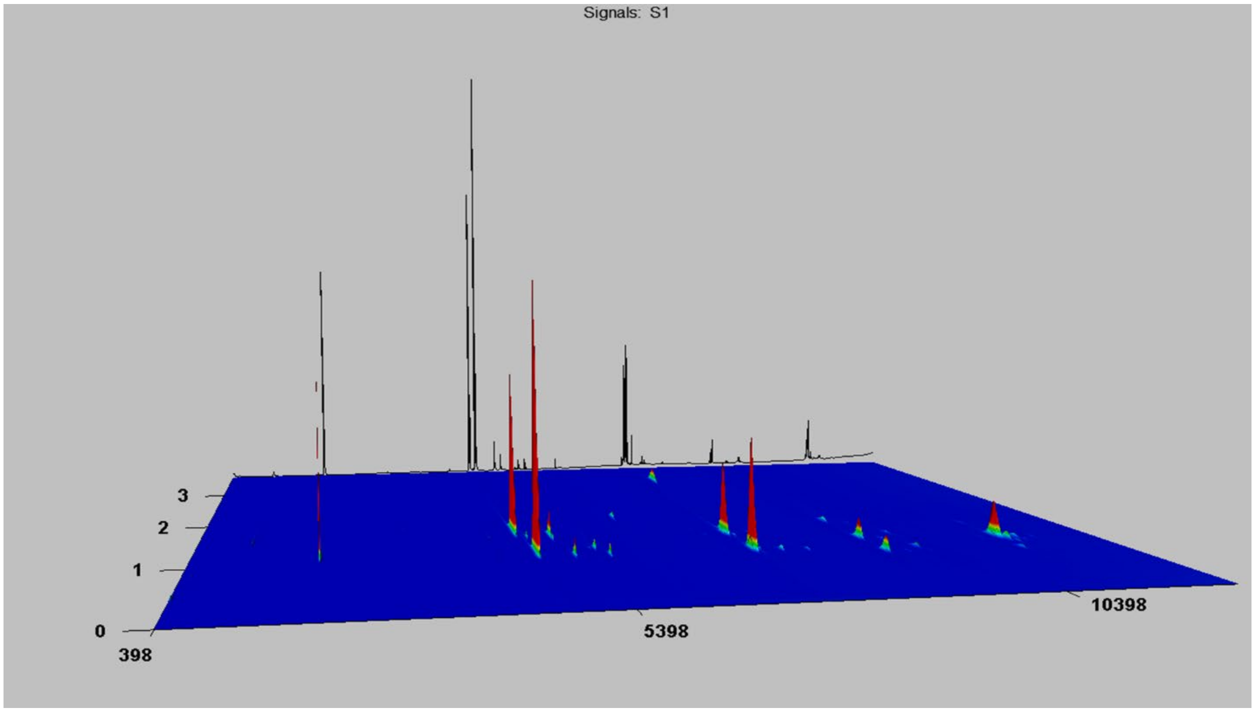

- Ru-II, *trans*-3-hexene, DCM, rt, 15 h.

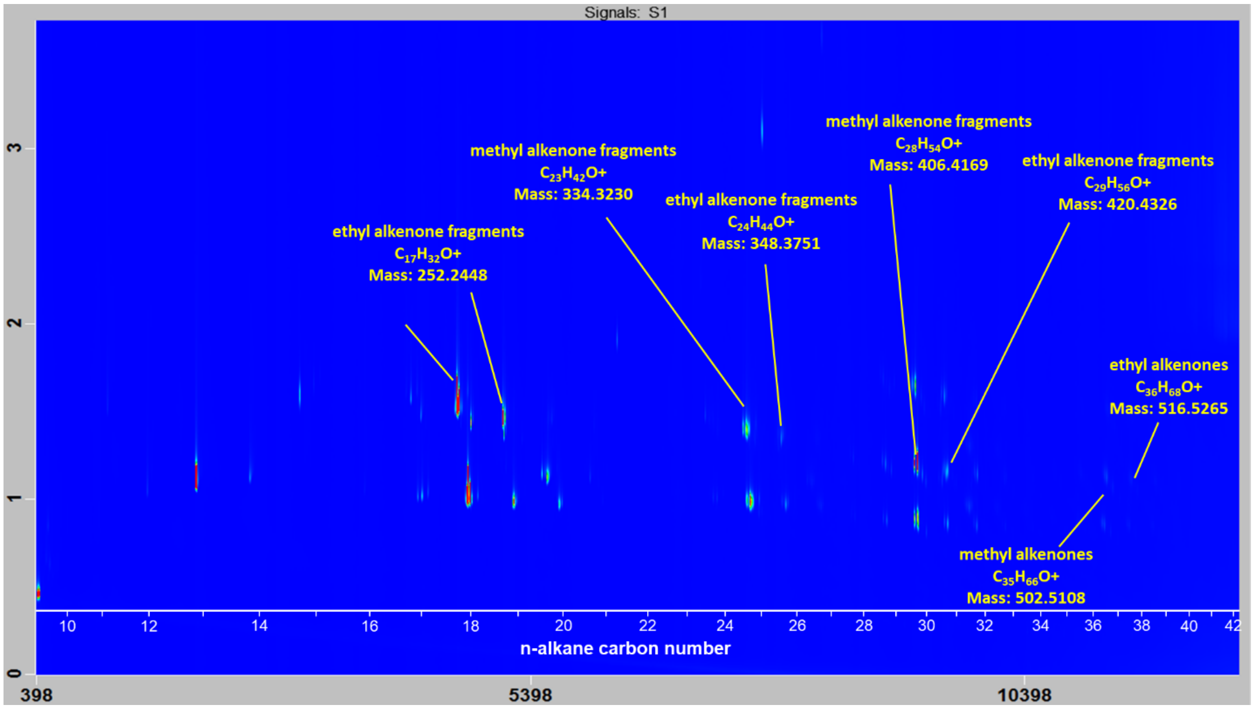

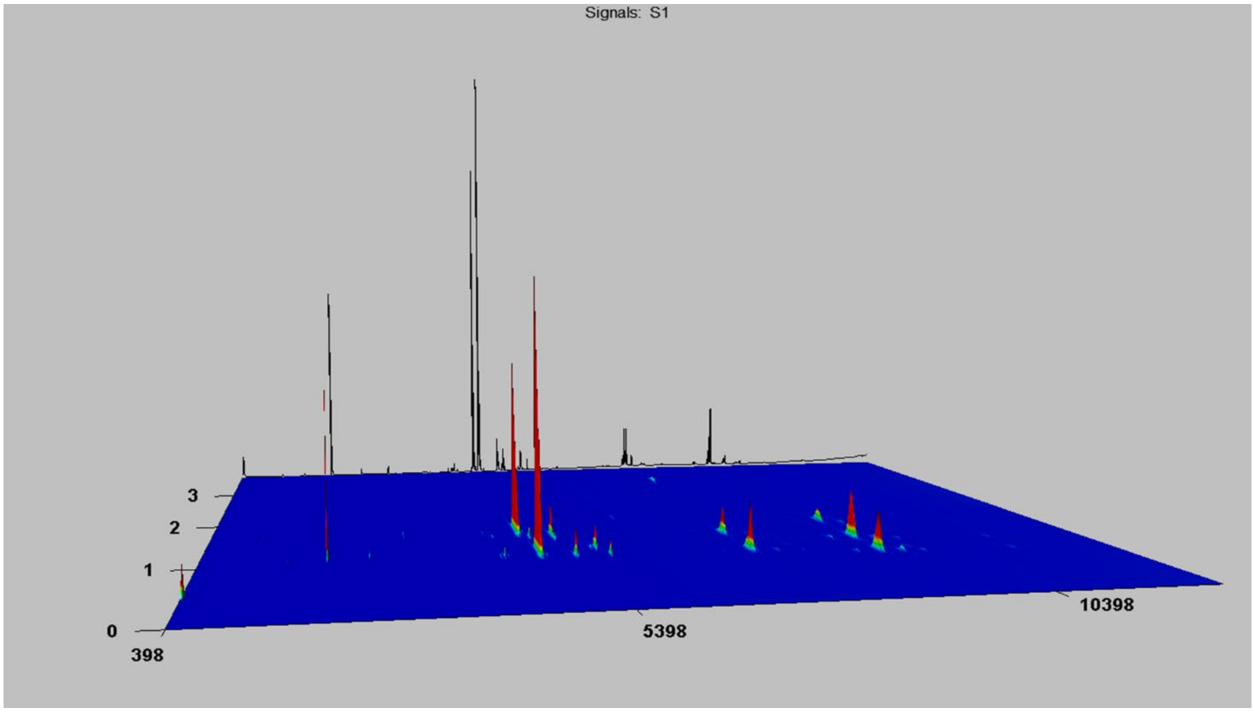

- Ru-II, *cis*-3-hexene, DCM, rt, 15 h.

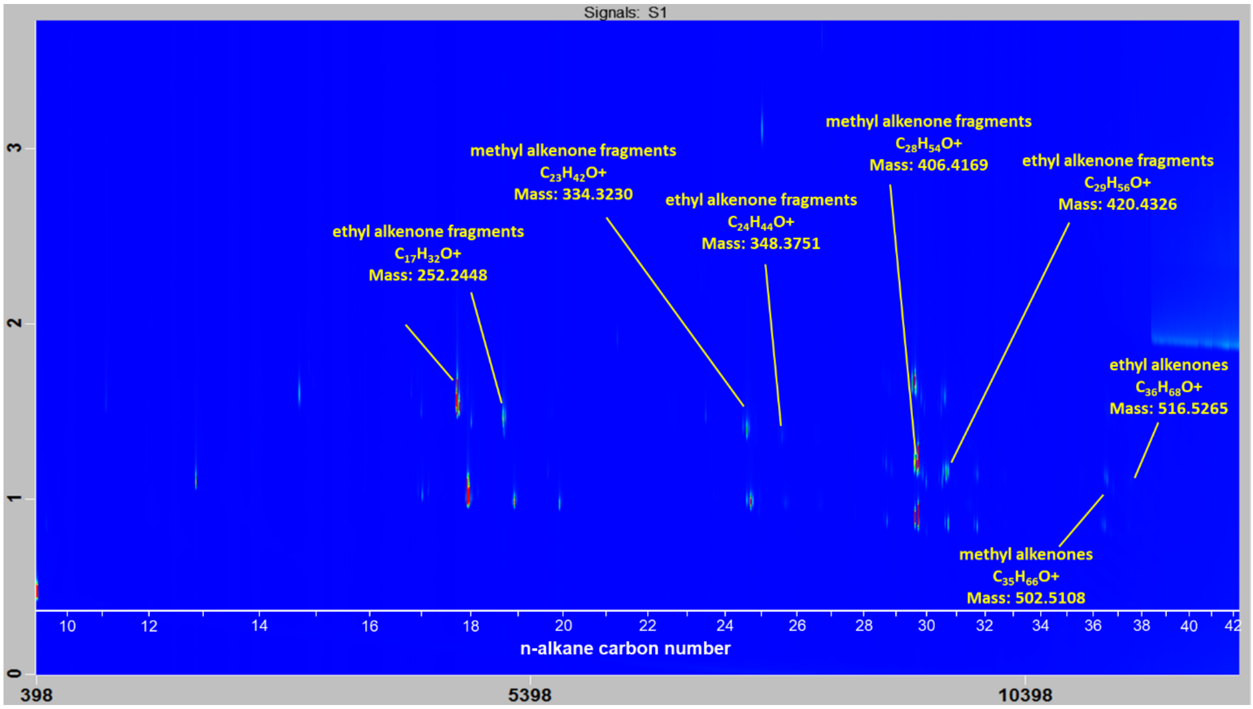

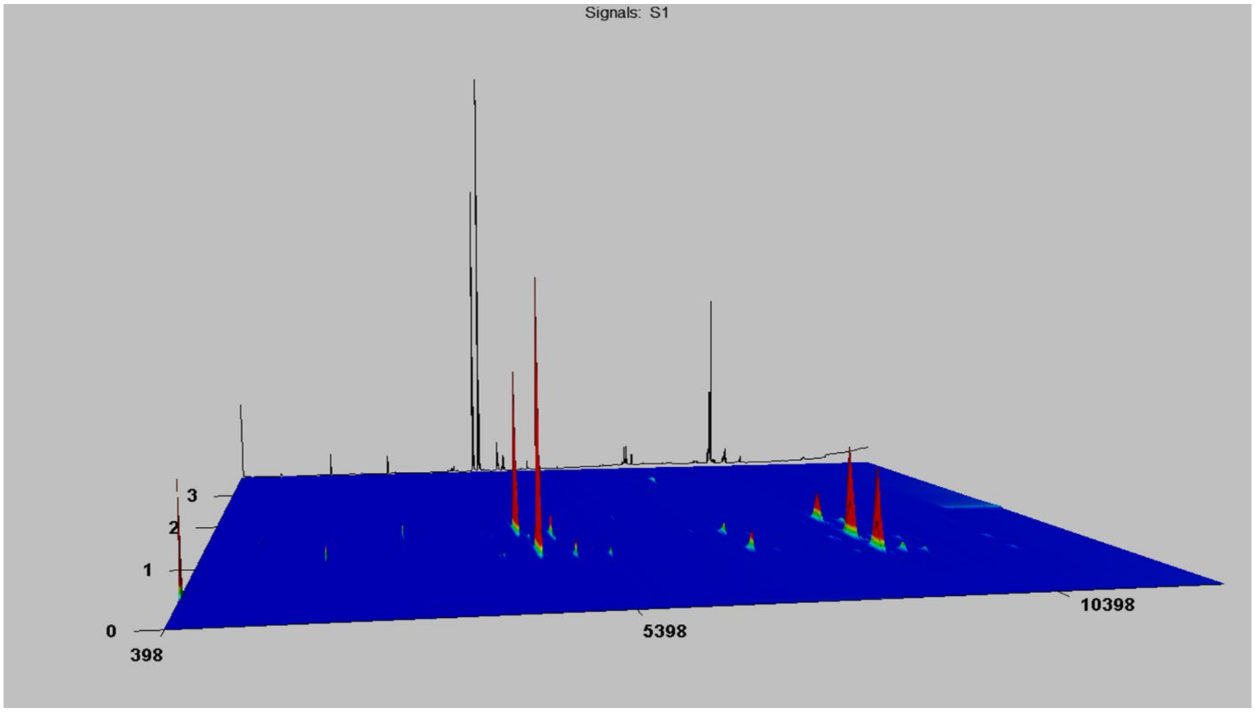

Figure S2. GC×GC-TOF high resolution mass spectra of alkenone hexenolysis products.

- Complete hexenolysis products:

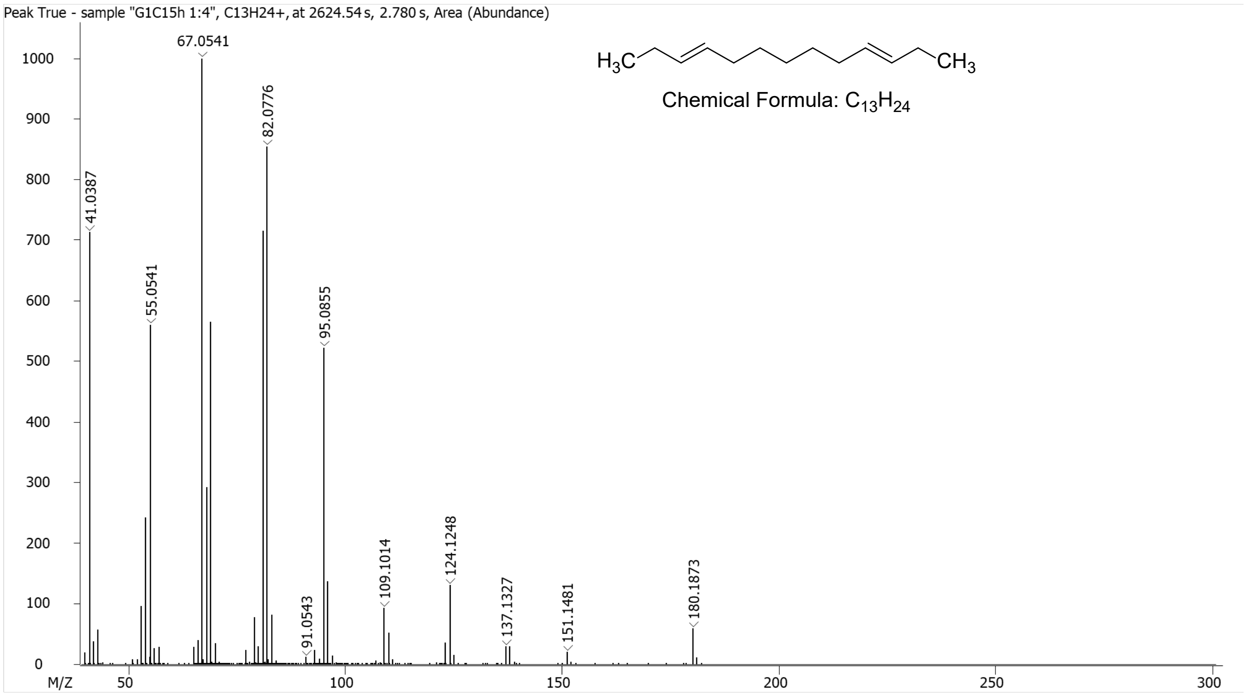

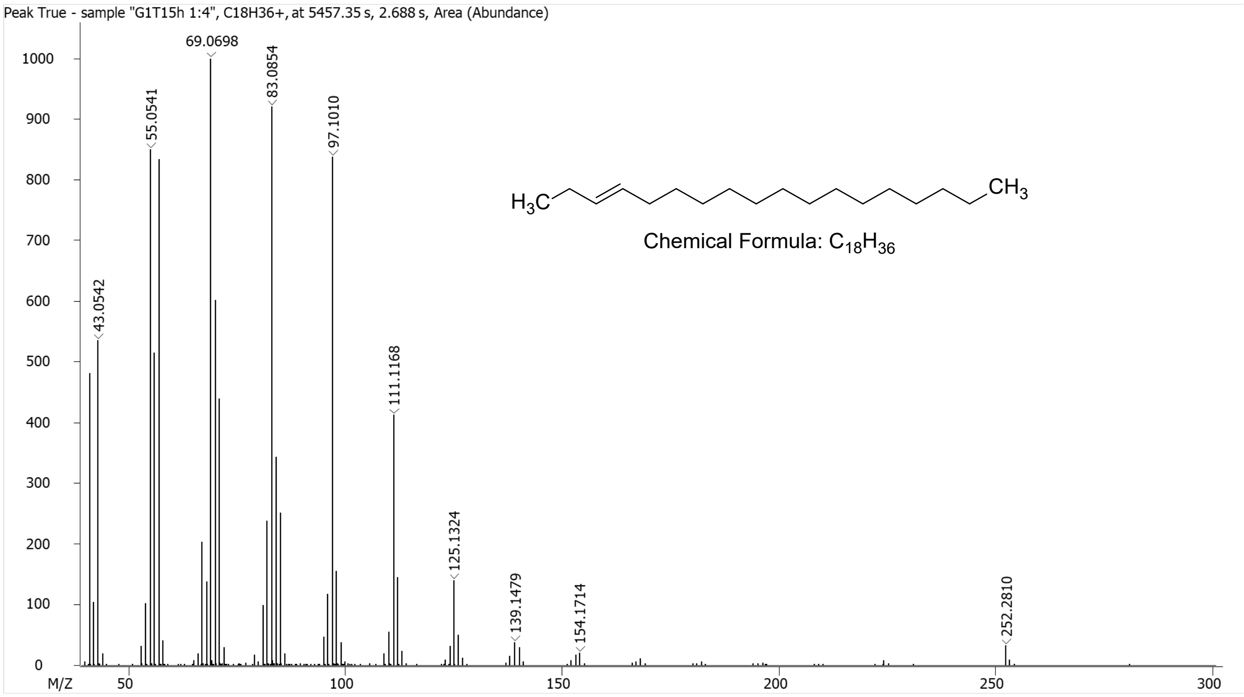

Peak True - sample "G1C15h 1:4", C17H32O+, at 5797.45 s, 3.738 s, Area (Abundance)

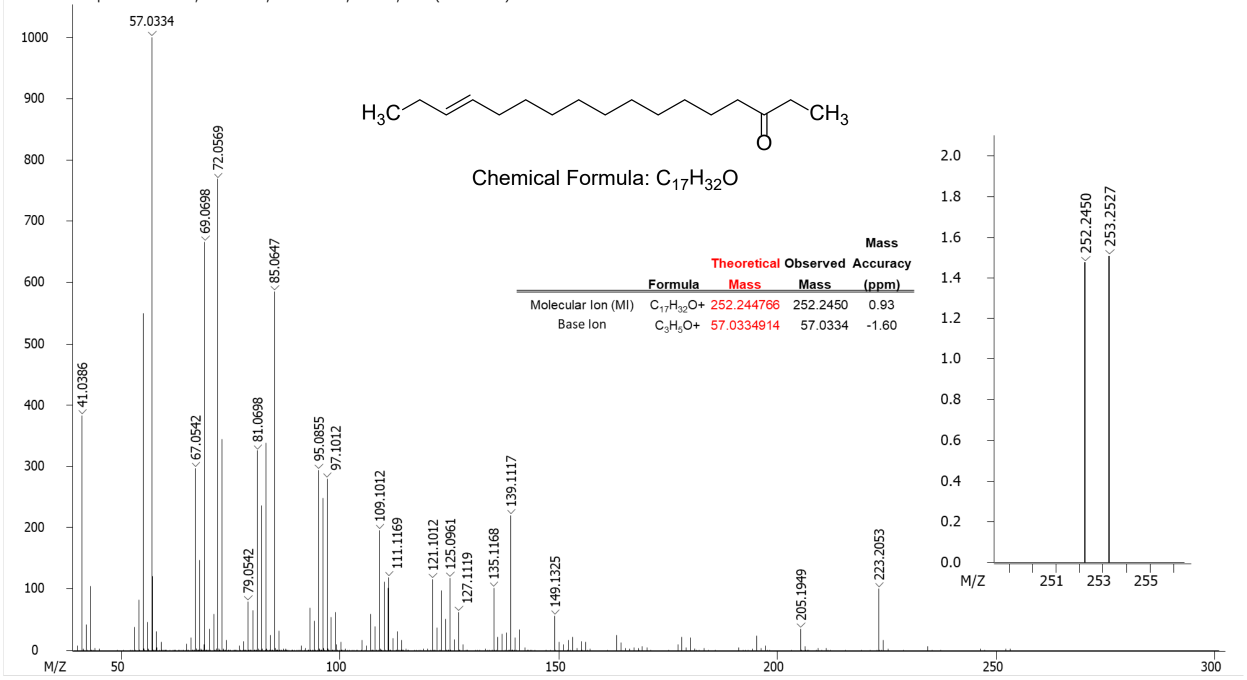

	Formula	Theoretical Mass	Observed Mass	Mass Accuracy (ppm)
Molecular Ion (MI)	$C_{17}H_{32}O+$	252.244766	252.2450	0.93
Base Ion	C_3H_5O+	57.0334914	57.0334	-1.60

Peak True - sample "G1C15h 1:4", C16H30O+, at 5325.32 s, 3.838 s, Area (Abundance)

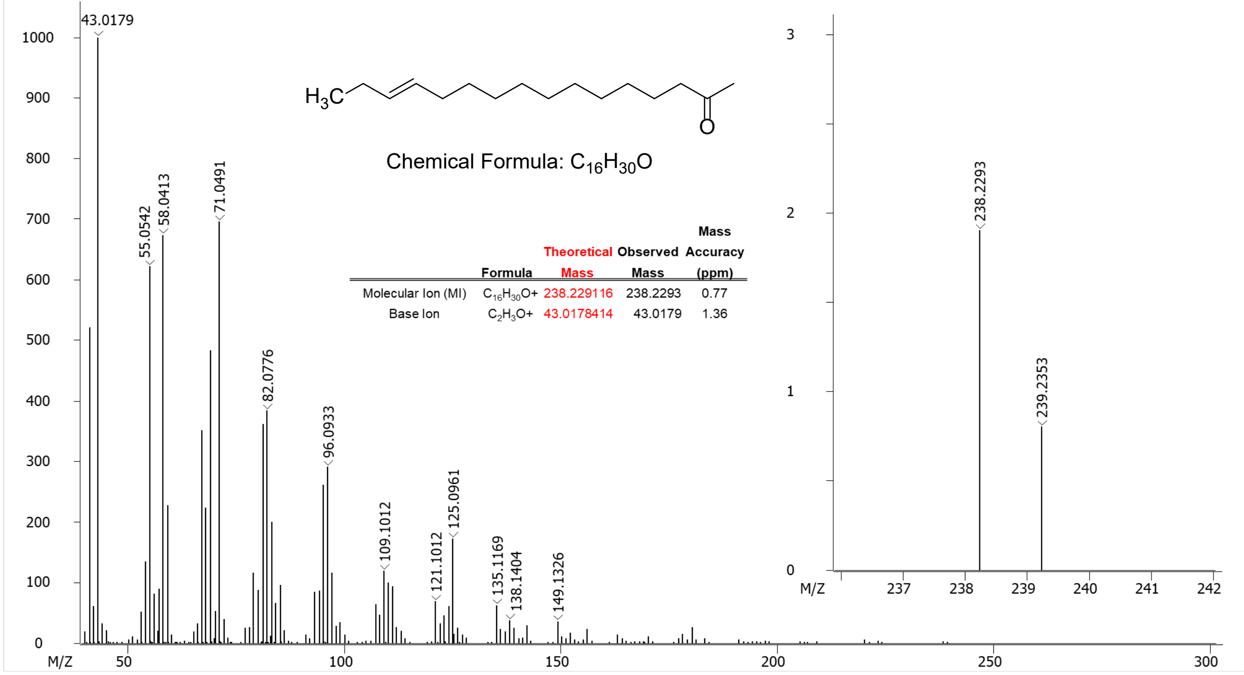

	Formula	Theoretical Mass	Observed Mass	Mass Accuracy (ppm)
Molecular Ion (MI)	$C_{16}H_{30}O+$	238.229116	238.2293	0.77
Base Ion	C_2H_3O+	43.0178414	43.0179	1.36

- Incomplete Hexenolysis Products:

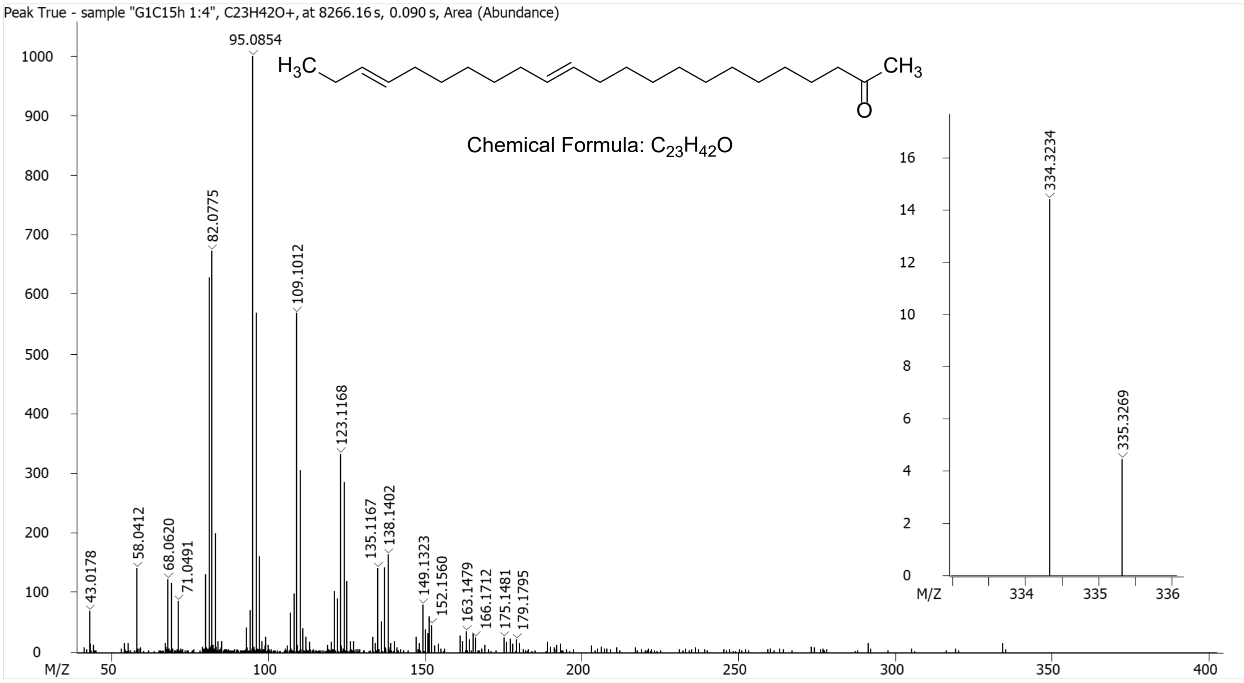

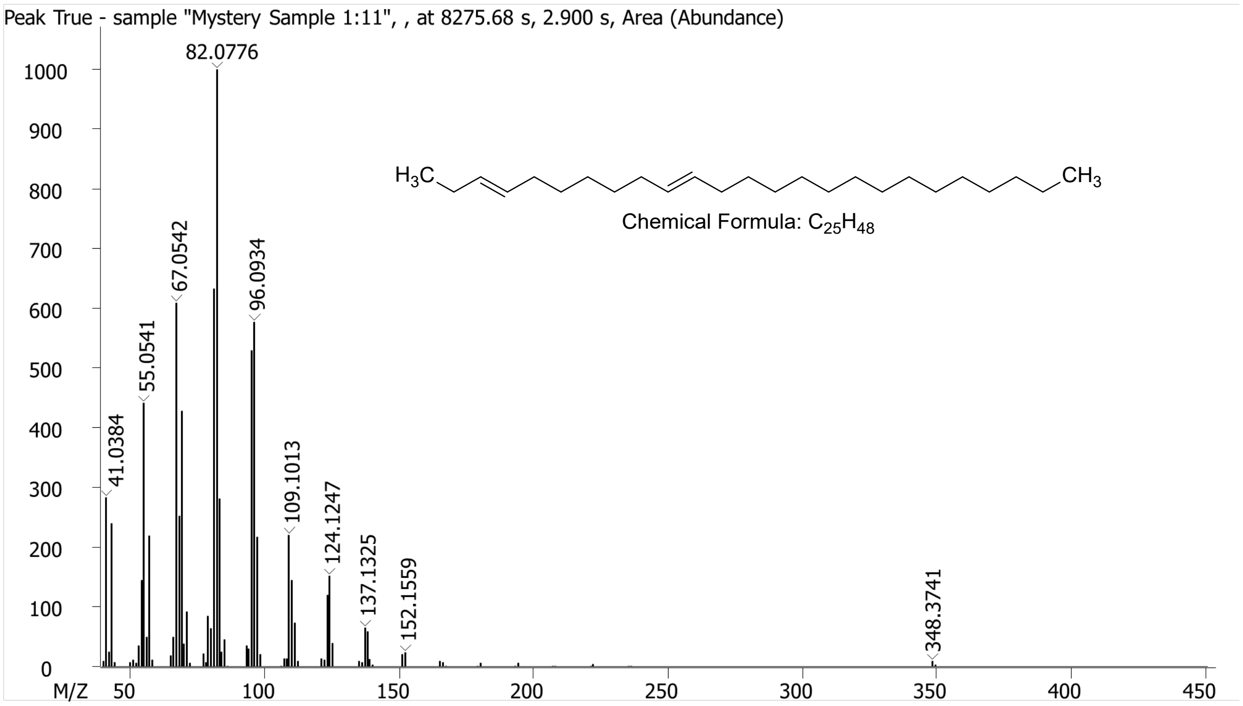

- Unexpected Hexenolysis Products:

Peak True - sample "G11C15h 1:4", C28H54O+, at 9910.63 s, 3.888 s, Area (Abundance)

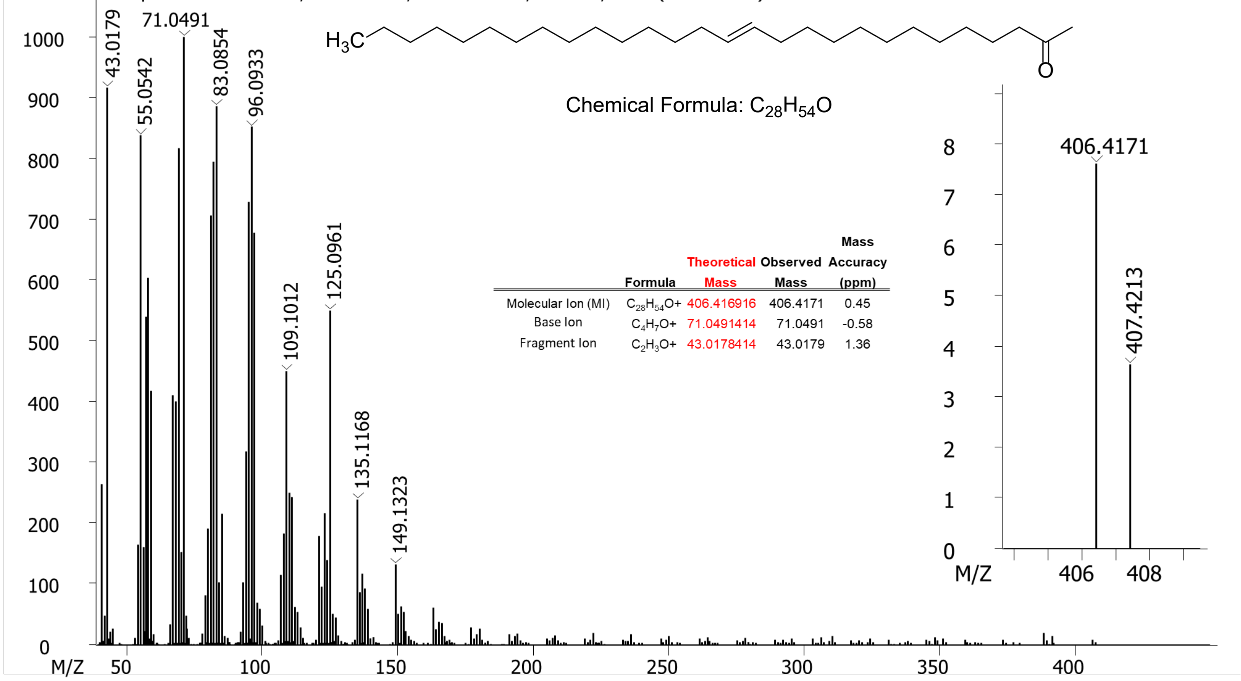

Peak True - sample "G1T5h 1:4", Peak 11, at 10226.7 s, 3.842 s, Area (Abundance)

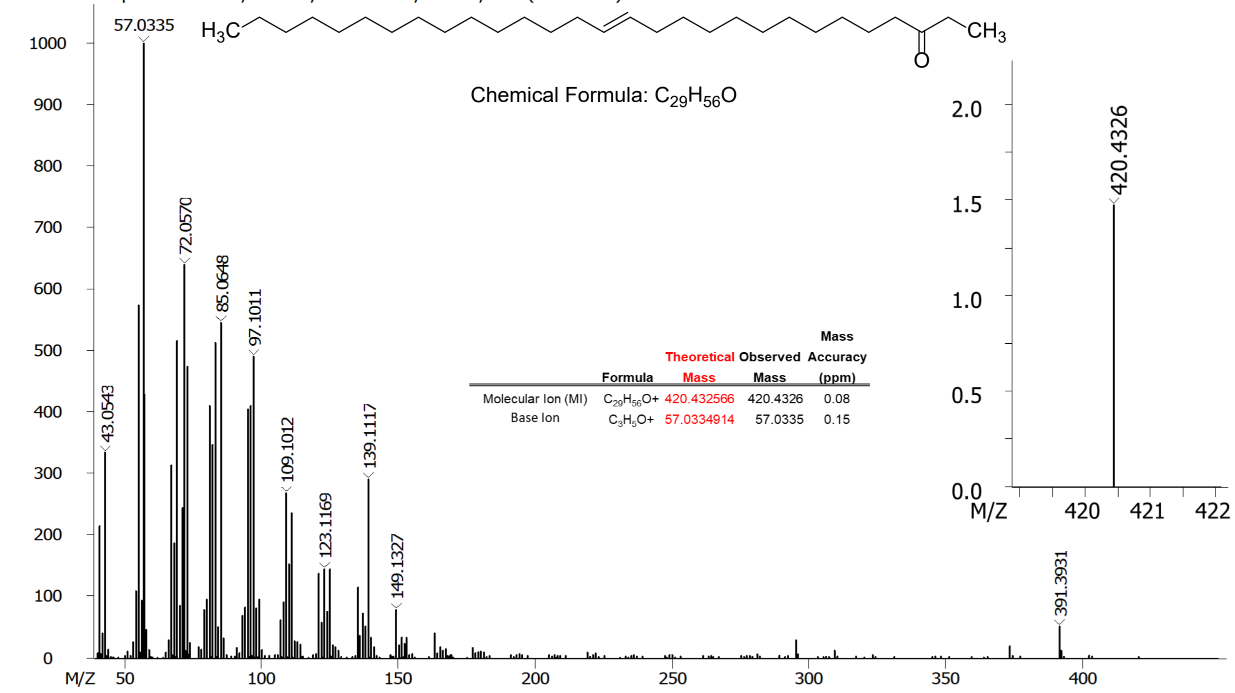

- Alkenones

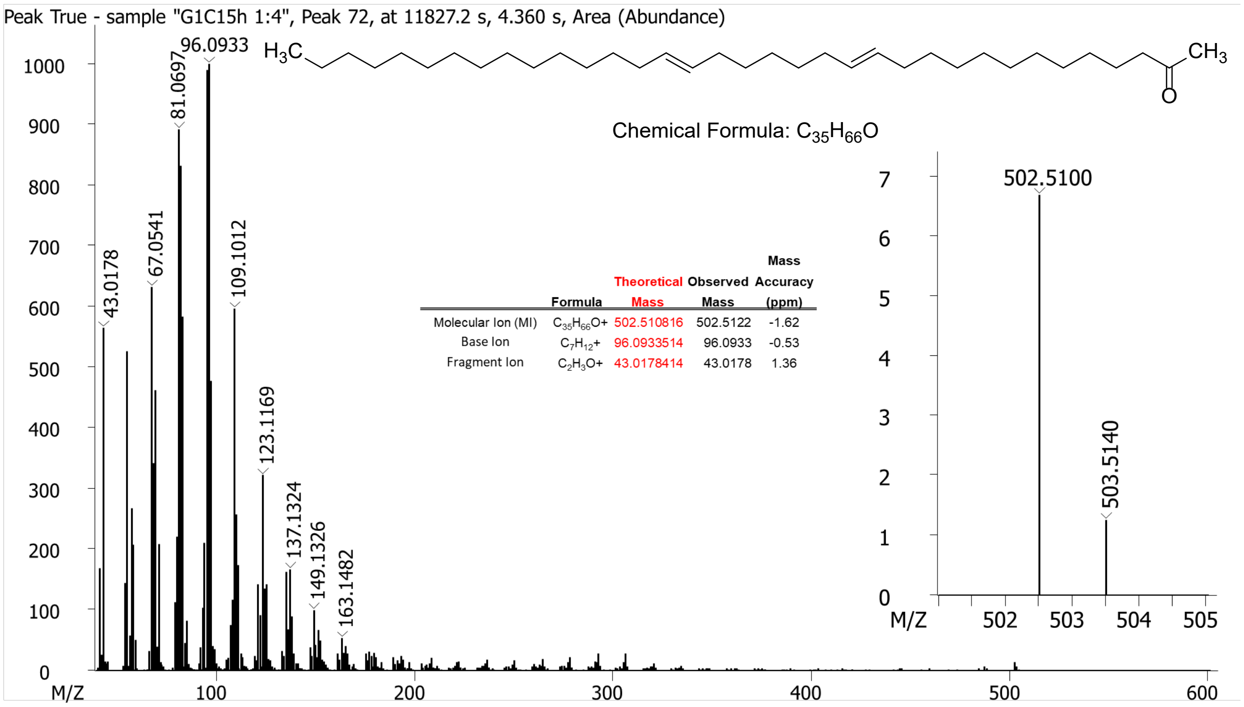

	Formula	Theoretical Mass	Observed Mass	Mass Accuracy (ppm)
Molecular Ion (MI)	$C_{35}H_{66}O+$	502.510816	502.5122	-1.62
Base Ion	$C_7H_{12}+$	96.0933514	96.0933	-0.53
Fragment Ion	C_2H_3O+	43.0178414	43.0178	1.36

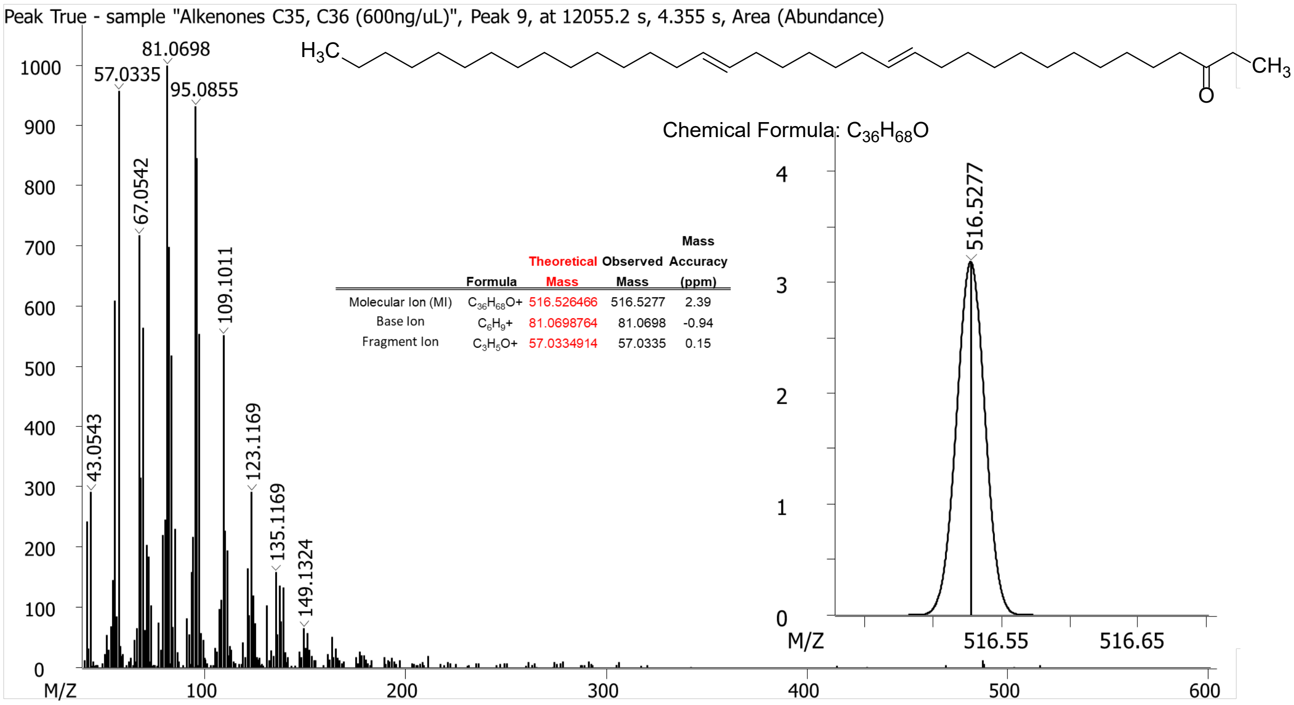

	Formula	Theoretical Mass	Observed Mass	Mass Accuracy (ppm)
Molecular Ion (MI)	$C_{36}H_{68}O+$	516.526466	516.5277	2.39
Base Ion	C_6H_9+	81.0698764	81.0698	-0.94
Fragment Ion	C_3H_5O+	57.0334914	57.0335	0.15

Figure S3. ^{1}H NMR Spectra of compounds 4, 5, and alkenone acrylate CM product mixtures.

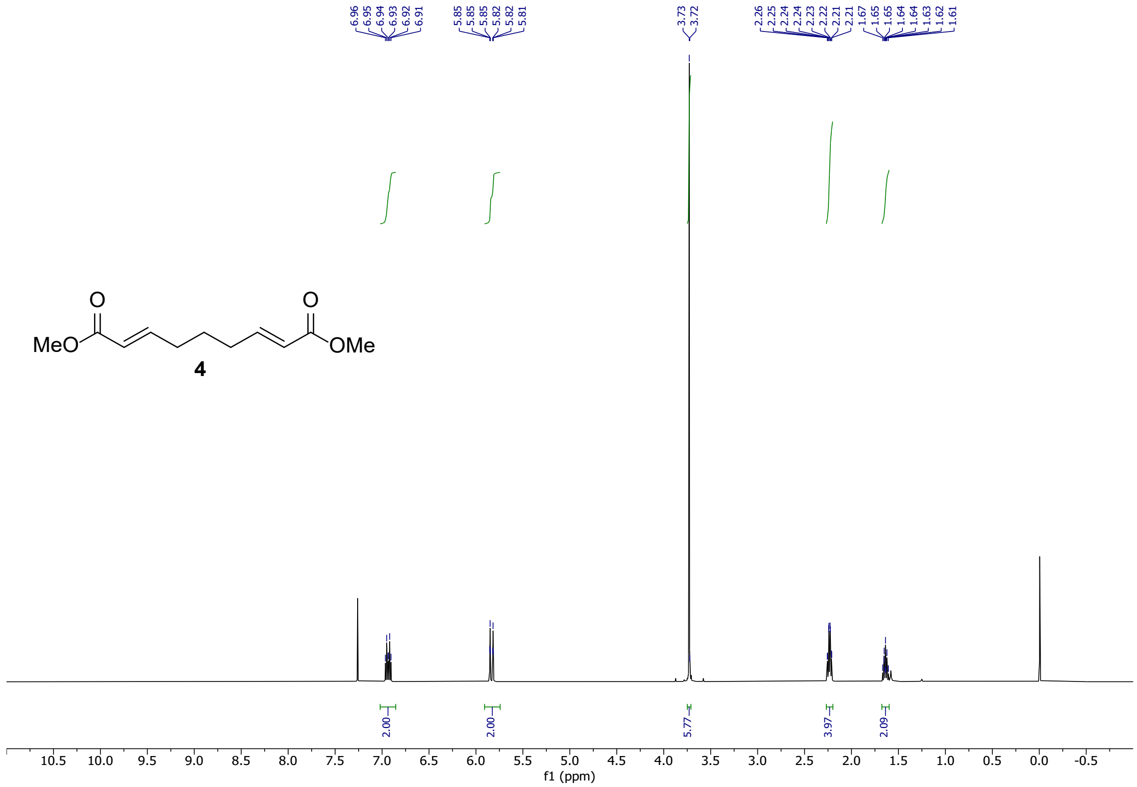

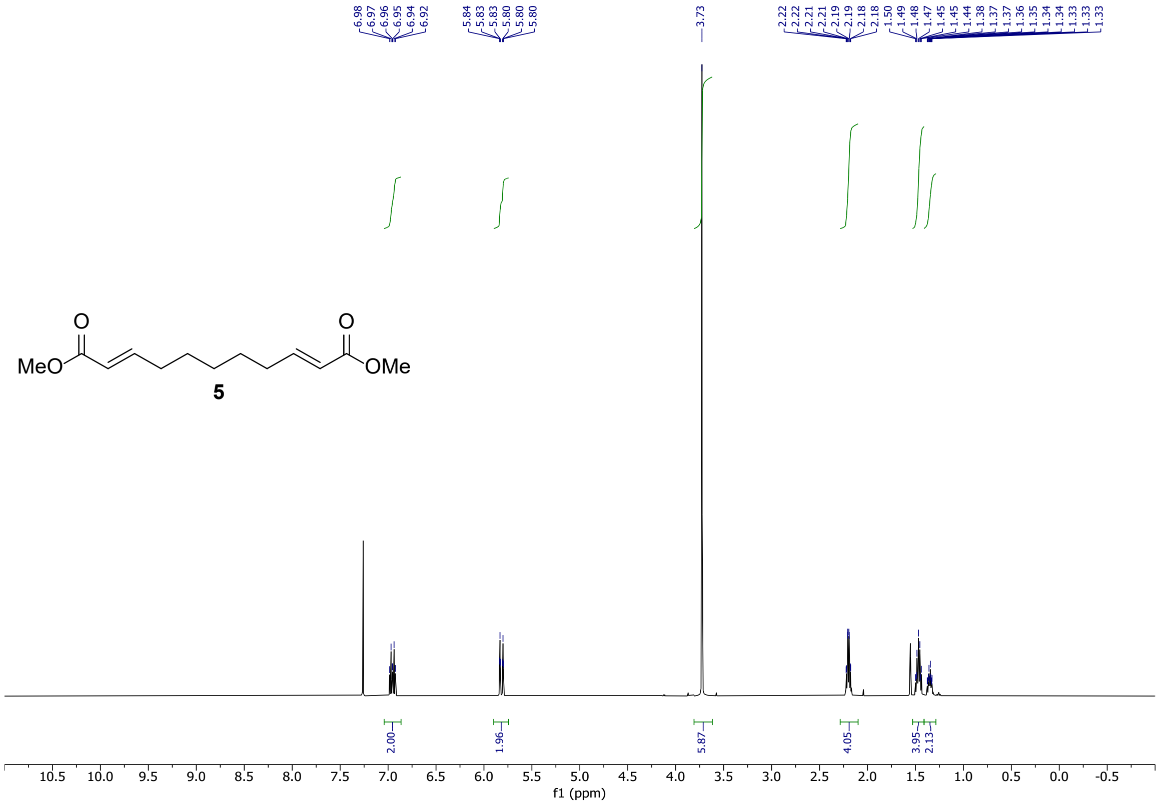

- 2021 *Phytobloom Tisochrysis* alkenone acrylate CM

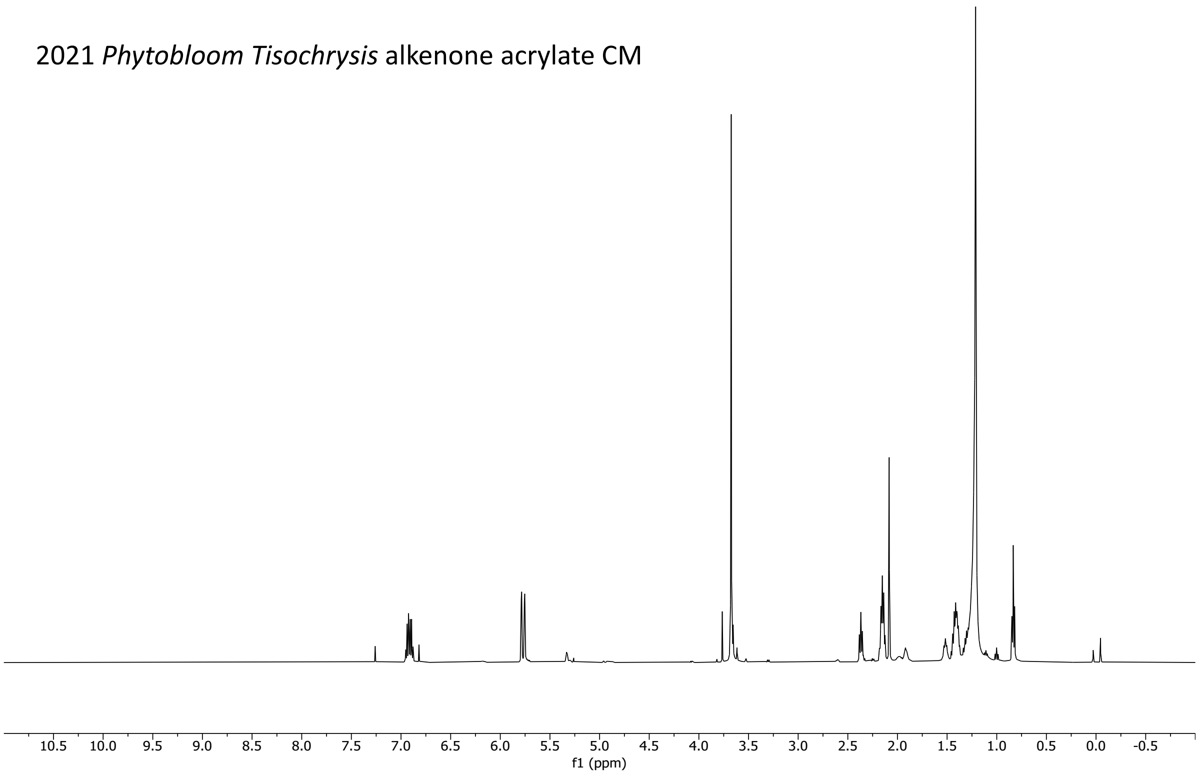

- 2016 *Phytobloom Tisochrysis* alkenone acrylate CM

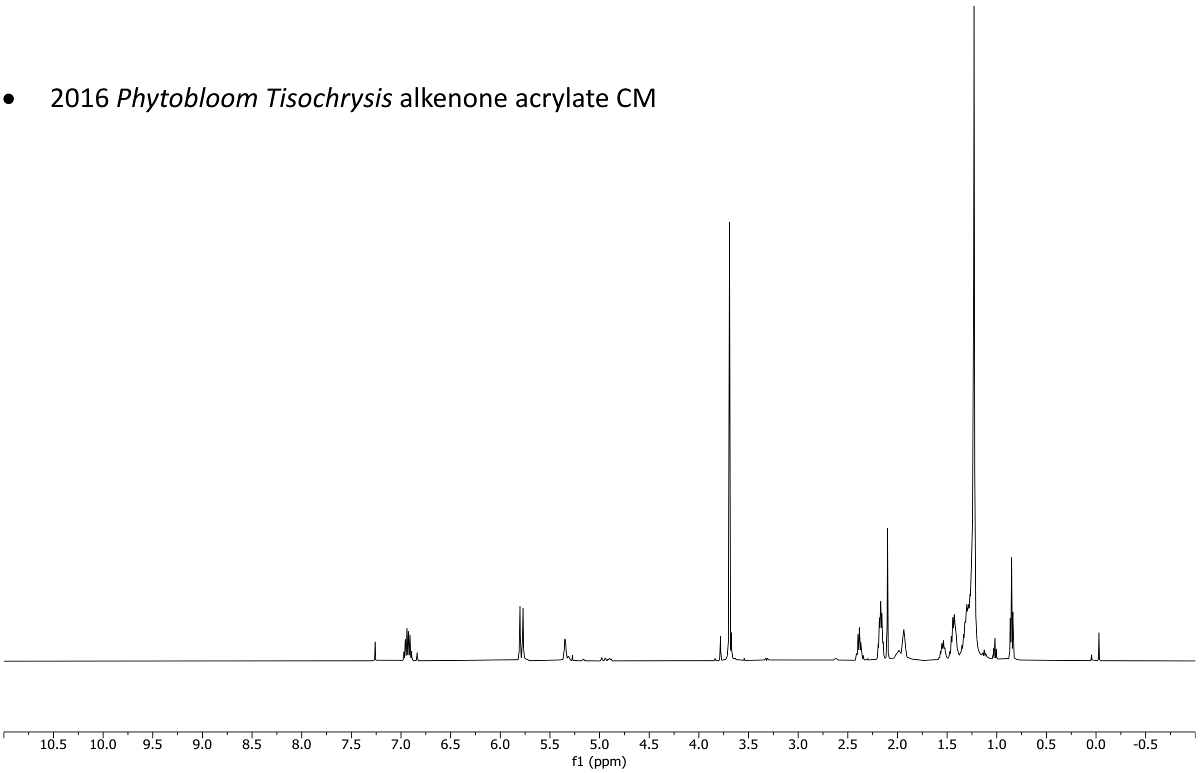

Supplement: Supplementary file 1 — ao4c00087_si_001.pdf [file ao4c00087_si_001.pdf]
